# Intestinal Ultrasonography for Predicting Postoperative Endoscopic Recurrence and Assessing Risk of Intestinal Surgery in Crohn’s Disease—A Systematic Review and Meta-Analysis of Prospective Studies

**DOI:** 10.3390/jcm15187024

**Published:** 2026-09-10

**Authors:** Murtaja Ahmad Alramahy, Batol Ahmad Alramahy, Jakob Benedict Seidelin, Panu Wetwittayakhlang, Jacob Tveiten Bjerrum, Mohamed Attauabi

**Affiliations:** 1 Department of Gastroenterology and Hepatology, Copenhagen University Hospital—Herlev and Gentofte, 2730 Herlev, Denmark; 2 Department of Clinical Medicine, Faculty of Health and Medical Sciences, University of Copenhagen, 2200 Copenhagen, Denmark; 3 Division of Gastroenterology, McGill University Health Centre, Montreal, QC H4A 3J1, Canada; 4 Gastroenterology and Hepatology Unit, Division of Internal Medicine, Faculty of Medicine, Prince of Songkla University, Songkhla 90110, Thailand

**Keywords:** intestinal ultrasonography, Crohn’s disease, postoperative recurrence, endoscopy, diagnostic accuracy, prediction, surgery

## Abstract

**Background/Objectives**: Postoperative endoscopic recurrence is common after intestinal resection for Crohn’s disease (CD), making surveillance essential. We performed a systematic review and meta-analysis evaluating the diagnostic performance of intestinal ultrasonography (IUS) for postoperative endoscopic recurrence and the prognostic association of IUS findings with surgical outcomes in the overall CD population. **Methods**: MEDLINE (PubMed) and Embase were searched from inception through 1 July 2026, for prospective studies evaluating postoperative IUS in CD. Diagnostic accuracy was analyzed using random-effects bivariate meta-analysis to estimate pooled sensitivity, specificity, likelihood ratios, summary receiver operating characteristic (SROC) curves, and diagnostic odds ratios (DORs). **Results**: Twenty-six prospective studies were included, comprising 797 patients evaluated for postoperative endoscopic recurrence and 1060 patients evaluated for associations between IUS findings and subsequent intestinal surgery. A bowel-wall thickness (BWT) threshold of >3.0 mm demonstrated high diagnostic accuracy for detecting any postoperative endoscopic recurrence (Rutgeerts ≥ i1), with pooled sensitivity of 85%, specificity of 83%, DOR of 30.8, and SROC AUC of 0.91, positive likelihood ratio of 5.3, and negative likelihood ratio of 0.19. For postoperative endoscopic recurrence (Rutgeerts ≥ i2), pooled sensitivity and specificity were lower at 79% and 67%, respectively (DOR 8.3; SROC AUC 0.76). A BWT threshold of >5.0 mm identified severe endoscopic recurrence (Rutgeerts i3–i4) with sensitivity of 79% and specificity of 81%, while a threshold of ≥7.0 mm was associated with increased risk of intestinal resections in the overall CD population. **Conclusions**: IUS demonstrated high diagnostic performance for postoperative endoscopic recurrence of CD with balanced sensitivity and specificity, and favourable likelihood ratios, although performance varied according to the definition and severity of recurrence. Evidence regarding diagnostic accuracy for modified Rutgeerts scores remains limited. Overall, these findings support IUS as a complementary modality to ileocolonoscopy, enabling repeated postoperative monitoring and risk stratification for endoscopic recurrence and surgery.

## 1. Introduction

Crohn’s disease (CD) is a chronic, transmural inflammatory disease that frequently progresses to strictures, fistulas, abscesses, and irreversible bowel damage requiring intestinal resection [1]. Despite advances in medical therapy, including immunomodulators, biological therapies, and small molecule therapies, approximately half of patients still require intestinal resections within 10 years of diagnosis [2,3].

Although surgery effectively treats complications, it is not curative, and postoperative endoscopic lesions and recurrence develop in 50–90% of patients within one year [4]. As recurrence correlates poorly with symptoms, early endoscopic surveillance is essential, with greater severity of recurrence predicting worse long-term outcomes [5,6]. The POCER trial established that treatment escalation guided by ileocolonoscopy using the Rutgeerts score at 6 months after ileocecal resection significantly reduced endoscopic recurrence, providing the evidence base for current international guidelines recommending surveillance ileocolonoscopy 6–12 months after surgery [5,6,7,8,9,10,11,12,13].

Intestinal ultrasonography (IUS) offers a non-invasive, radiation-free, and well-tolerated alternative for repeated assessment of postoperative CD [14]. Ultrasonographic features, including bowel-wall thickness (BWT), bowel-wall stratification, vascularity, and transmural complications, correlate with postoperative Rutgeerts score and may provide additional transmural and structural information beyond mucosal assessment, and have therefore been proposed as surrogate markers of postoperative endoscopic recurrence [15]. However, the available evidence remains heterogeneous in terms of ultrasound techniques, diagnostic thresholds, recurrence definitions, and timing of assessment, making it difficult to draw firm conclusions regarding its diagnostic performance. Therefore, we performed a systematic review and meta-analysis to evaluate the diagnostic accuracy of IUS for detecting postoperative endoscopic recurrence according to clinically relevant Rutgeerts definitions, and to determine the prognostic association of IUS findings with subsequent surgical outcomes.

## 2. Materials and Methods

This systematic review and meta-analysis was conducted in accordance with the Preferred Reporting Items for Systematic Reviews and Meta-Analyses of Diagnostic Test Accuracy Studies (PRISMA-DTA) guidelines (Supplementary Material Table S1) [16] and followed methodological guidance from the Cochrane guidelines [17]. The protocol was registered in PROSPERO prior to study initiation (registration number: 420261427042).

### 2.1. Search Strategy

At least two independent authors systematically searched MEDLINE (PubMed) and Embase from database inception to 1 July 2026, aiming to identify studies evaluating the diagnostic accuracy of IUS for postoperative endoscopic recurrence, as well as studies assessing the association between IUS findings and subsequent clinical or surgical outcomes in CD, including primary intestinal resections and re-resections. The search strategy combined controlled vocabulary and free-text terms related to CD, intestinal or bowel ultrasonography, prognosis, recurrence, treatment outcomes, and pre-operative or postoperative assessment (Supplementary Material Table S2). In addition, reference lists of relevant reviews were screened manually to identify additional eligible studies. An English-language restriction was applied, and only full-text reports were eligible for inclusion.

Study selection was performed independently by at least two reviewers in two stages, comprising title and abstract screening followed by full-text assessment. Disagreements were resolved by consensus or, when necessary, consultation with a third reviewer.

### 2.2. Eligibility Criteria

Eligible studies included patients with a confirmed diagnosis of CD and prospectively evaluated transabdominal IUS in relation to pre- or post CD-related resections. Eligible study designs comprised prospective cohort studies, case–control studies, diagnostic cross-sectional studies with prospectively acquired index and reference tests, randomized controlled trials, and relevant analyses nested within clinical trials. No maximum interval between IUS and ileocolonoscopy was prespecified in the review protocol. Eligible ultrasound techniques included conventional B-mode IUS, colour Doppler IUS, oral-contrast ultrasonography (OCUS), small intestine contrast ultrasonography (SICUS), contrast-enhanced ultrasonography (CEUS), and composite IUS scores or algorithms. Studies including pediatric or mixed-age populations were eligible. Eligible studies were grouped according to either diagnostic accuracy analyses in terms of endoscopic recurrence or prognostic analyses of future intestinal surgery. In the latter, IUS findings were evaluated in relation to both primary resections and re-resections. Studies without data suitable for quantitative synthesis were retained for narrative synthesis when they provided relevant diagnostic or prognostic evidence. Studies were classified as concurrent diagnostic accuracy studies when IUS and ileocolonoscopy were performed within the same diagnostic episode, and as forward-prediction studies when an earlier IUS examination was used to predict findings at ileocolonoscopy.

Reviews, editorials, letters, conference abstracts, unpublished data, case reports, retrospective studies, and studies combining CD with other conditions without separately extractable CD data were excluded.

### 2.3. Outcomes

The pre-defined primary outcome was the diagnostic accuracy of IUS for detecting postoperative endoscopic recurrence. Because clinically distinct endoscopic definitions exist, the original and modified Rutgeerts classifications were analyzed separately according to predefined thresholds: (1) any endoscopic recurrence (Rutgeerts ≥ i1); conventional endoscopic recurrence (Rutgeerts ≥ i2); recurrence according to the modified Rutgeerts classification (modified Rutgeerts ≥ i2b); and severe endoscopic recurrence (Rutgeerts i3–i4).

Secondary outcomes included diagnostic performance according to IUS modality, BWT threshold, and timing of assessment, associations with endoscopic severity, and the prognostic association between IUS findings and risk of intestinal surgery. Prognostic outcomes were classified separately as surgical recurrence in postoperative cohorts and future intestinal surgery in broader CD populations.

### 2.4. Data Extraction

At least two authors independently extracted data using a predefined Microsoft Excel collection form. Extracted variables included study and patient characteristics, outcomes, IUS modality, IUS operator experience, blinding to ileocolonoscopy findings, scanning protocols, interobserver reliability, anatomical assessment site, index-test definitions and thresholds, reference standards, timing of IUS, endoscopy, and follow-up, as well as reported outcomes. Specific endoscopic and IUS scores were extracted separately. Where available, true-positive (TP), false-positive (FP), false-negative (FN), and true-negative (TN) values were extracted or reconstructed. Separate estimates for different modalities, thresholds, outcomes, and assessment time points were also extracted. The inequality symbols used for BWT thresholds (> or ≥) were reported as defined in the original publications, and the term “approximately” was used to combine these inequality symbols in pooled analyses.

### 2.5. Risk of Bias

Risk of bias was assessed independently by at least two authors using QUADAS-2 for diagnostic accuracy studies, which evaluates bias related to patient selection, the index test, the reference standard, and flow and timing. Each domain was classified as having low, high, or unclear risk of bias [18]. For forward-prediction and prognostic-factor studies, the Quality In Prognosis Studies tool (QUIPS) was used to assess risk of bias across six domains: study participation, study attrition, prognostic-factor measurement, outcome measurement, study confounding, and statistical analysis and reporting. Studies contributing to both research questions were assessed separately using the corresponding tool for each analysis [19].

### 2.6. Statistical Analysis

Statistical analyses were performed in R version 4.6.0 using the metafor and mada packages [20,21], according to the endoscopic recurrence definition, IUS modality, and BWT threshold. Log diagnostic odds ratios (log DORs) were pooled using random-effects models, and heterogeneity was assessed using Cochran’s Q, $I^{2}$, and $\tau^{2}$. A continuity correction of 0.5 was applied to studies containing a zero cell [22]. Bivariate random-effects models were used to estimate pooled sensitivity and specificity and to generate summary receiver operating characteristic (SROC) curves, likelihood ratios, DORs, and areas under the curves (AUCs). Differences between conventional transabdominal IUS and OCUS were explored using bivariate random-effects meta-regression. For intestinal surgery, unadjusted odds ratios were calculated, and comparable studies were pooled; other IUS predictors were presented individually. The relationship between time from surgery to IUS and the time between IUS and ileocolonoscopy and diagnostic performance was evaluated exploratively with random-effects meta-regression. Effects on sensitivity and false-positive rate were modelled jointly and evaluated using likelihood-ratio tests. Further, the relationship between the IUS-to-ileocolonoscopy interval and diagnostic performance was explored in a sensitivity analysis restricting assessments to those conducted within 30 days.

## 3. Results

### 3.1. Study Characteristics

The systematic search identified 1612 records, while 67 additional records were identified through screening of existing reviews, resulting in a total of 1679 records. Of these, 26 studies met the eligibility criteria and were included in the systematic review (Supplementary Material Figure S1). Seventeen studies (797 patients) evaluated IUS for the detection of postoperative endoscopic recurrence [23,24,25,26,27,28,29,30,31,32,33,34,35,36,37,38,39], seven studies (1060 patients) evaluated associations between IUS findings and future intestinal surgery in broader CD populations [40,41,42,43,44,45,46], and two studies (212 patients) evaluated longitudinal postoperative symptomatic and/or surgical recurrence and were synthesized separately [47,48]. Of the postoperative endoscopic recurrence studies, 11 (575 patients) contributed to the grouped diagnostic accuracy meta-analysis [26,27,28,29,30,32,33,34,36,37,39], whereas all seven surgery-prediction studies contributed to the prognostic meta-analysis [40,41,42,43,44,45,46].

Postoperative cohorts included 17-108 predominantly adult patients (reported age range, 16–86 years; female proportion, 25.0–64.1%). Median disease duration ranged from 3.8 to 12 years. Most patients underwent ileocecal, ileal, or ileocolonic resection with an ileocolonic anastomosis (ICA) [23,24,25,26,27,28,29,30,31,32,33,34,35,36,37,38,39], and two cohorts also included stricturoplasty in addition to resection [26,28]. When specified, postoperative BWT was assessed at the ICA, perianastomotic region, and/or neoterminal ileum (Table 1 and Table 2). IUS was performed in a period of three months to 30 years postoperatively; concurrent IUS-ileocolonoscopy intervals ranged from same-day assessment to 6 months, while forward-prediction intervals reached 12 months [25,27,29,36,37]. Conventional IUS predominated [23,24,25,26,28,30,33,35,36,37,38,39], while colour Doppler [30,33,35,36,37,38,39], oral-contrast IUS [27,28,29,31,32,34], and CEUS were also evaluated [33,36] (Table 1 and Table 2). Apart from two studies which evaluated endoscopic recurrence according to the modified Rutgeerts classification [35,38], all studies defined postoperative endoscopic recurrence using the original Rutgeerts score. Operator experience and technical conduct were variably reported (Supplementary Table S3). Several studies used highly experienced operators [23,26,30,31,32,38], and blinding to ileocolonoscopy findings was reported in many diagnostic studies [23,24,26,27,28,30,31,35,37,38,39]. Scanning protocols varied substantially, while formal IUS interobserver reliability was rarely assessed; Allocca et al. reported excellent interobserver agreement (κ = 0.84) [35] (Supplementary Table S3).

Overall, QUADAS-2 assessments classified nine studies as low risk [23,24,26,27,28,35,36,37,38], four studies as having some concerns [29,31,32,34], and three studies as high risk [30,33,39], most frequently due to the index-test [31,33,39] and flow-and-timing domains [29,30,32,36,39]. QUIPS assessment of prognostic-factor studies classified five studies as high risk of bias [25,42,43,44,45], five for moderate risk of bias [40,41,46,47,48] and none as low risk of bias. (Supplementary Material Tables S4 and S5). High risk-of-bias ratings were primarily driven by concerns regarding study confounding [25], study participation [43,44], and analysis and reporting [42,43,45], while all studies rated as having moderate risk of bias had concerns in the study confounding, analysis, and reporting domains [40,41,46,47,48].

### 3.2. Any Postoperative Endoscopic Recurrence

**Figure 1:**
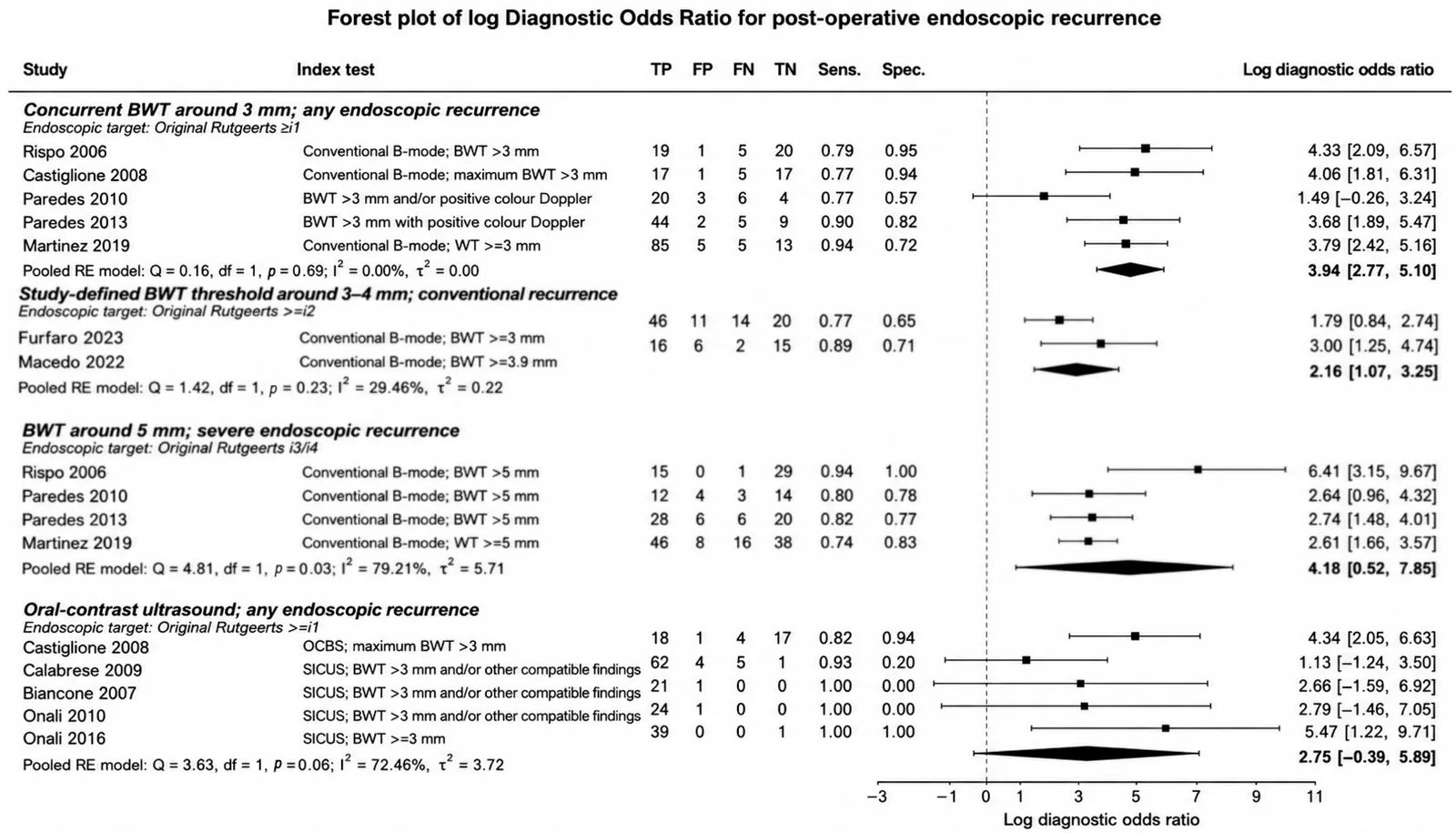
Diagnostic odds ratio for specific IUS BWT thresholds and endoscopic recurrence definitions. TP, true positive; FN, false negative; FP, false positive; TN, true negative; Sens., sensitivity; Spec, specificity; BWT, bowel-wall thickness; RE, random-effect.

**Figure 2:**
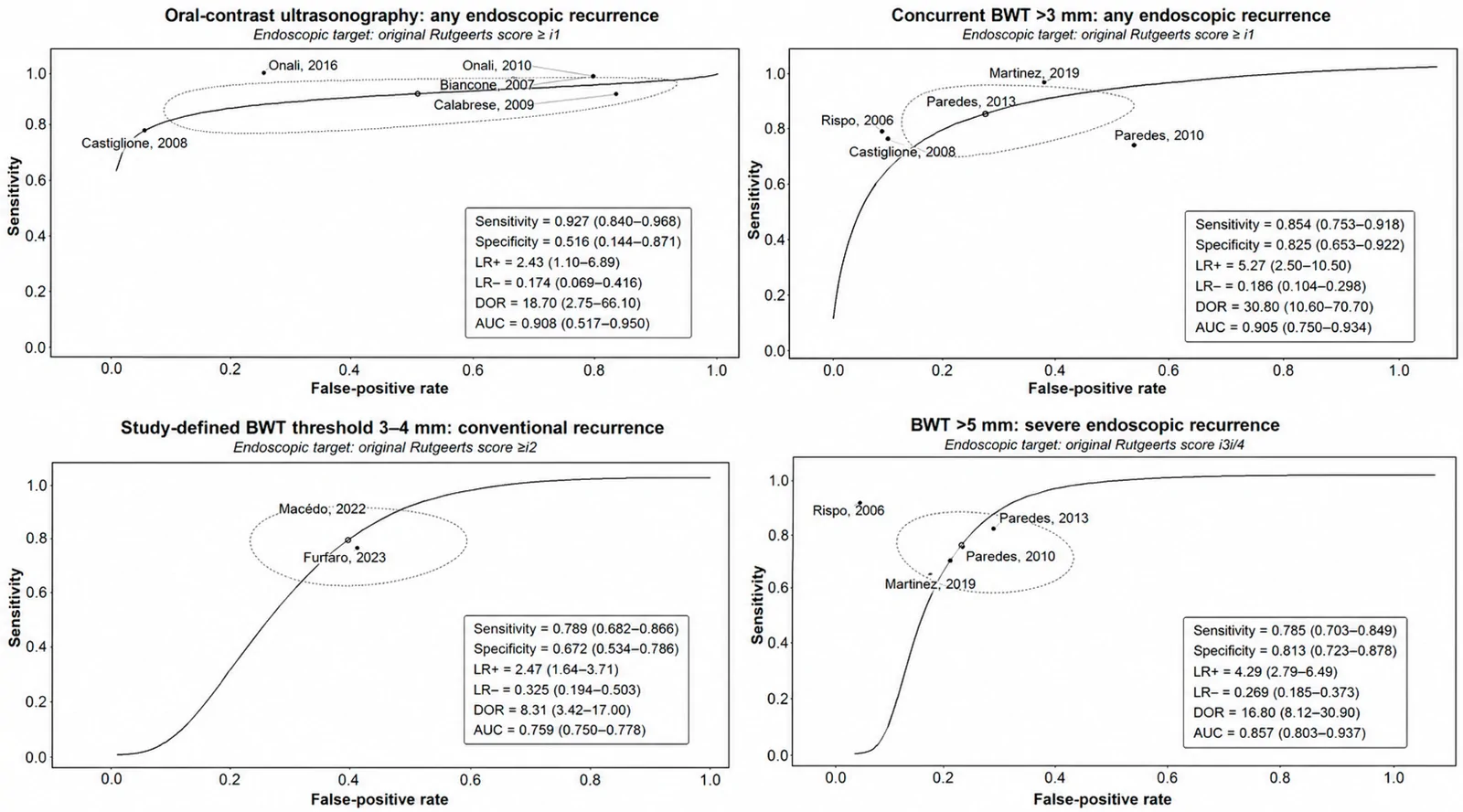
Summary receiver operating characteristic (SROC) curves for postoperative intestinal ultrasonography according to BWT threshold. LR+, positive likelihood ratio; LR-, negative likelihood ratio; DOR, diagnostic odds ratio; AUC, area under the curve; BWT, bowel-wall thickness.

Main diagnostic performance measures are summarized in Table 3. Concurrent transabdominal IUS using a BWT threshold of >3.0 mm demonstrated high diagnostic accuracy for detecting any postoperative endoscopic recurrence (Rutgeerts ≥ i1) [26,28,30,33,36]. Across five studies comprising 286 patients, pooled sensitivity was 0.85 (95% CI, 0.75–0.92) and specificity was 0.83 (95% CI, 0.65–0.92), corresponding to a DOR of 30.80 (95% CI, 10.60–70.70) and an area under the SROC curve of 0.91 (95% CI, 0.75–0.93), with no evidence of statistical heterogeneity (I^2^ = 0%) (Figure 1 and Figure 2). The positive likelihood ratio was 5.27 (95% CI, 2.50–10.50) and the negative likelihood ratio was 0.19 (95% CI, 0.10–0.30).

Diagnostic performance varied according to endoscopic severity. Conventional IUS with BWT > 3.00 mm detected 93% of severe (Rutgeerts i3/i4) recurrence but only 38% of mild (Rutgeerts i1/i2) recurrence in the study by Castiglione et al. [28]. Similarly, five of six false-negative examinations with this BWT threshold in Paredes et al. represented mild endoscopic recurrence [30], whereas four of six false-negative examinations in Andreoli et al. involved lesions located predominantly on the colonic side of the anastomosis [24].

Two additional studies evaluated higher BWT thresholds (>5 mm). Andreoli et al. reported sensitivity of 81% and specificity of approximately 89% among 41 patients for detecting any postoperative endoscopic recurrence (Rutgeerts ≥ i1) [24], whereas DiCandio et al. reported sensitivity of 82% and specificity of 100% when using a composite radiological-endoscopic reference standard rather than the Rutgeerts classification [23]. Also, with this BWT threshold, false-negative examinations in both studies represented early asymptomatic recurrence.

### 3.3. Conventional Endoscopic Recurrence

For conventional postoperative endoscopic recurrence (Rutgeerts ≥ i2), two studies comprising 130 patients evaluated BWT thresholds between 3 and 4 mm [37,39]. Pooled sensitivity was 0.79 (95% CI, 0.68–0.87) and specificity was 0.67 (95% CI, 0.53–0.79), corresponding to an LR+ of 2.47 (95% CI, 1.64–3.71) and an LR− of 0.33 (95% CI, 0.19–0.50). The estimated SROC area under the curve was 0.76 (95% CI, 0.75–0.78). The pooled DOR was 8.31 (95% CI, 3.42–17.00), with low-to-moderate heterogeneity (I^2^ = 29.5%) (Figure 1 and Figure 2). As only two studies were included in the meta-analysis, pooled estimates should be considered exploratory.

Furfaro et al. (91 patients) found that BWT ≥ 3.0 mm alone yielded a sensitivity of 0.77 (95% CI, 0.64–0.87) and specificity of 0.65 (95% CI, 0.45–0.81), whereas combining BWT ≥ 3.0 mm with fecal calprotectin ≥ 50 μg/g reduced sensitivity to 0.65 (95% CI, 0.51–0.78) but increased specificity to 0.93 (95% CI, 0.76–0.99) [39]. Each 1 mm increase in BWT was independently associated with endoscopic recurrence (adjusted odds ratio, aOR, 2.43; 95% CI, 1.21–4.89), while mesenteric lymph nodes were highly specific (0.97) but insensitive (0.35) and independently associated with recurrence (aOR, 15.63; 95% CI, 1.48–164.54) [39]. Similarly, Macedo et al. (39 patients) identified an ROC-derived optimal BWT threshold of 3.9 mm, yielding sensitivity of 89% and specificity of 71% [37]. An ordinal IUS severity score outperformed clinical assessment and inflammatory biomarkers, achieving an AUROC of 0.82.

### 3.4. Severe Postoperative Endoscopic Recurrence

For severe postoperative endoscopic recurrence (Rutgeerts i3/i4), four studies comprising 246 patients evaluated a BWT threshold of ≥5 mm [26,30,33,36]. Pooled sensitivity was 0.79 (95% CI, 0.70–0.85) and specificity was 0.81 (95% CI, 0.72–0.88 corresponding to an LR+ of 4.29 (95% CI, 2.79–6.49) and an LR− of 0.27 (95% CI, 0.19–0.37). The pooled DOR was 16.80 (95% CI, 8.12–30.90), and the SROC area under the curve was 0.86 (95% CI, 0.80–0.94), with substantial between-study heterogeneity (I^2^ = 79.2%) (Figure 1 and Figure 2).

### 3.5. Additional Ultrasound Modalities and Factors Potentially Influencing Diagnostic Performance

#### 3.5.1. Oral-Contrast Ultrasonography

**Figure 3:**
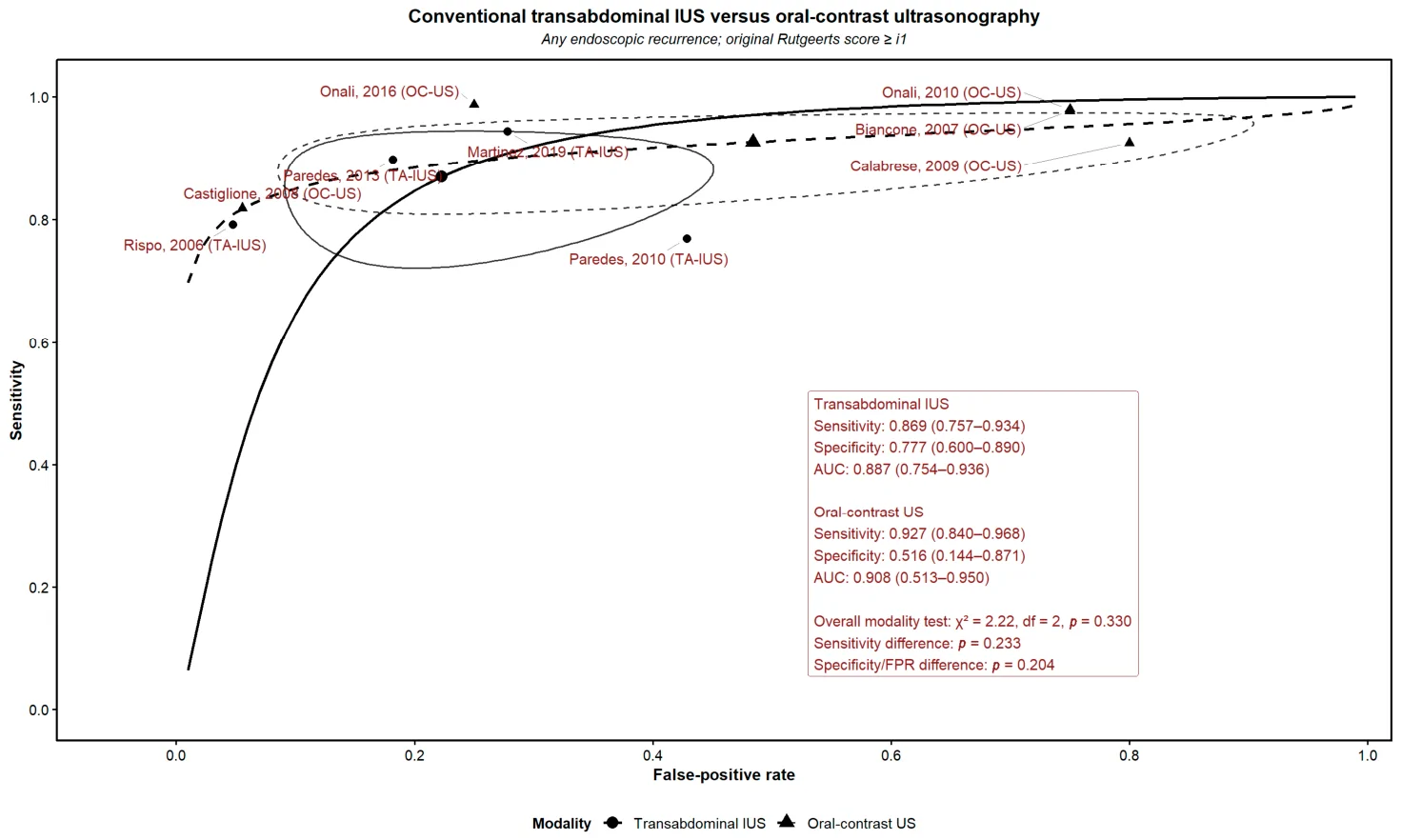
Diagnostic accuracy of conventional transabdominal IUS versus oral-contrast ultrasonography. *IUS, intestinal ultrasonography; AUC, area under the curve.*

Five studies, comprising 199 patients, evaluated OCUS for detecting any postoperative endoscopic recurrence (Rutgeerts ≥ i1) [27,28,29,32,34], yielding a pooled sensitivity of 0.93 (95% CI, 0.84–0.97), whereas specificity was 0.52 (95% CI, 0.14–0.87), corresponding to an area under the SROC curve of 0.91 (95% CI, 0.52–0.95). The pooled DOR was 18.70 (95% CI, 2.75–66.10, I^2^ = 72.5%) (Figure 1 and Figure 3).

Only one study (40 patients) directly compared conventional transabdominal IUS with OCUS [28], reporting a modest increase in sensitivity (77% vs. 82%) without loss of specificity (94% for both modalities), although the difference was not statistically significant [28]. Consistent with this finding, exploratory meta-regression demonstrated no significant differences between conventional and OCUS in overall diagnostic performance (*p* = 0.330), sensitivity (*p* = 0.233), or specificity (*p* = 0.204) (Figure 3).

Longitudinal evidence for SICUS was limited. In a subgroup of 10 patients, Biancone et al. reported SICUS findings compatible with recurrence in four patients (40%) at 3 months, eight (80%) at 6 months, and all 10 (100%) at 12 months [27]. In a separate cohort by Onali et al., BWT measured by SICUS at 1 year was not significantly higher in patients who subsequently developed clinical relapse at 2 years than in those who remained in remission (median, 5.0 vs. 3.7 mm; *p* = 0.19), whereas the 1-year endoscopic score was significantly higher among patients who subsequently relapsed (median, 4 vs. 2; *p* = 0.003) [32].

In the study by Paredes et al. (60 patients), a composite CEUS-based rule comprising neoterminal-ileal BWT > 5.0 mm or bowel-wall contrast enhancement > 46% detected any postoperative endoscopic recurrence (Rutgeerts ≥ i1) with a sensitivity of 96% and specificity of 100% [33]. In the Martínez et al. study, requiring both BWT ≥ 3.0 mm and bowel-wall contrast enhancement ≥ 46% did not improve accuracy for any postoperative endoscopic recurrence (Rutgeerts ≥ i1) compared with BWT ≥ 3.0 mm alone, although a separate composite model showed better performance than BWT ≥ 5.0 mm alone for severe endoscopic recurrence (Rutgeerts i3–i4; sensitivity, 90%; specificity 87%) [36].

#### 3.5.2. Timing of Postoperative IUS

Evidence regarding the optimal timing of postoperative IUS was limited, with only one study providing forward-prediction data of 50 patients [25]. The study performed IUS at three months and ileocolonoscopy at 12 months after surgery. Although BWT ≥ 5.0 mm demonstrated high specificity (90.0%) for endoscopic recurrence, sensitivity was only 26.3%, with 14 of 19 recurrent cases missed by the early ultrasound examination at three months [25]. In a longitudinal subgroup of 10 patients, Biancone et al. reported SICUS-positive findings in four (40.0%) patients at 3 months, eight (80.0%) at 6 months, and all 10 (100%) at 12 months [27].

**Figure 4:**
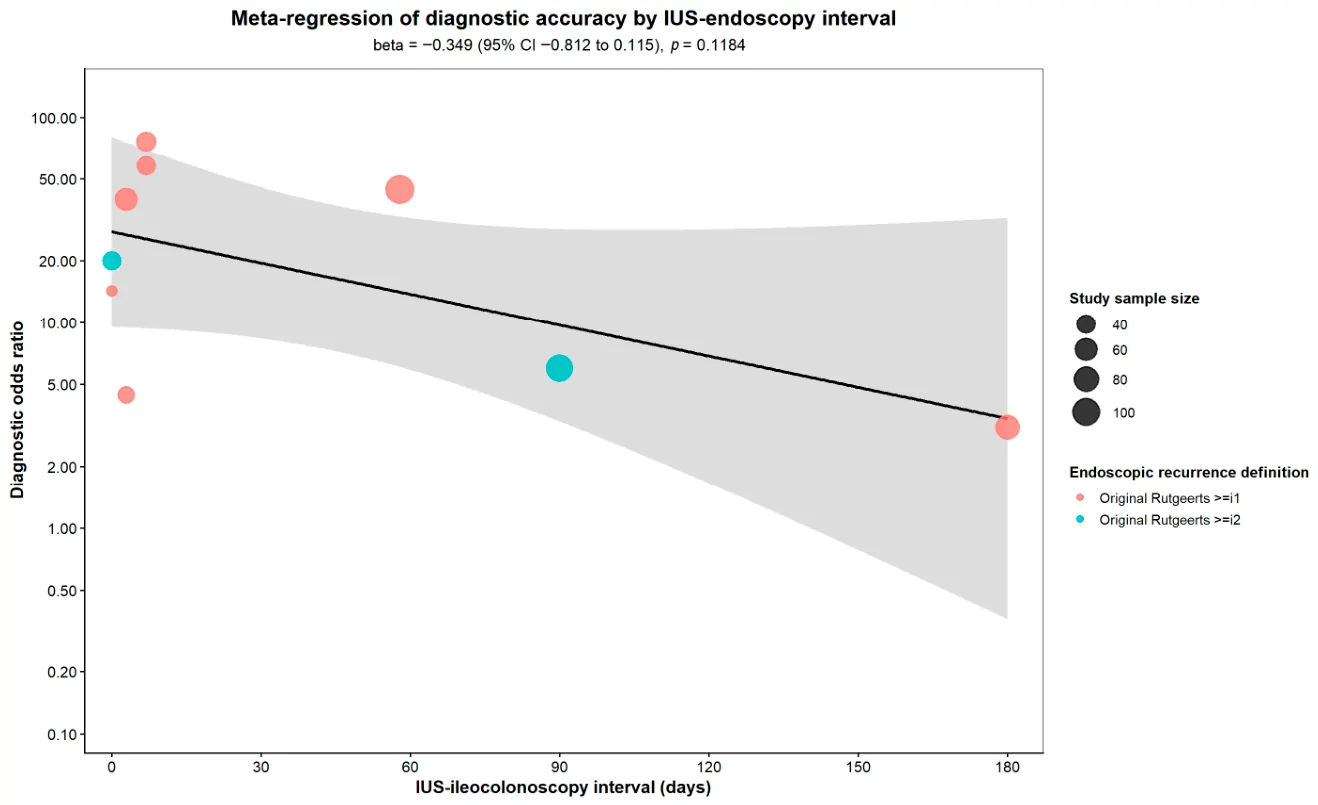
Exploratory meta-regression for the association between diagnostic odds ratio and time between IUS and endoscopy. *IUS, intestinal ultrasonography.*

In exploratory meta-regression analysis comparing IUS with ileocolonoscopy for endoscopic recurrence according to Rutgeerts score, diagnostic performance was not significantly associated with time from surgery to IUS (DOR ratio 0.92 (95% CI, 0.70–1.20); χ^2^ = 1.32; df = 2; *p* = 0.52), with no change in sensitivity (OR, 0.88; 95% CI, 0.68–1.13) nor specificity (OR, 1.04; 95% CI, 0.76–1.44) [26,27,28,29,30,32,33,34,36,37]. Similarly, in a meta-regression of nine studies, the interval between IUS and ileocolonoscopy was not associated with overall diagnostic performance (DOR ratio 0.71 (95% CI, 0.50–1.05; *p* = 0.085; χ^2^ = 6.00; df = 2; *p* = 0.12), with no change in sensitivity (OR, 1.07; 95% CI, 0.84–1.36; *p* = 0.58); however, specificity decreased (OR, 0.68; 95% CI, 0.52–0.89; *p* = 0.006) (Figure 4) [26,27,28,29,30,33,36,37,39].

In sensitivity analyses restricted to studies in which IUS and ileocolonoscopy were performed within 30 days, four studies comprising 178 patients were included for any postoperative endoscopic recurrence (Rutgeerts ≥ i1) using a BWT threshold of approximately 3 mm [26,28,30,33], yielding a pooled sensitivity of 0.82 (95% CI, 0.74–0.88) and specificity of 0.85 (95% CI, 0.62–0.95). For conventional postoperative endoscopic recurrence (Rutgeerts ≥ i2), only one study comprising 39 patients met the 30-day criterion [37], with a sensitivity of 89% and specificity of 71%, precluding meta-analysis. For severe postoperative endoscopic recurrence (Rutgeerts i3–i4) using a BWT threshold of approximately 5 mm, three studies comprising 138 patients yielded a pooled sensitivity of 0.84 (95% CI, 0.72–0.91) and specificity of 0.80 (95% CI, 0.67–0.89) [26,30,33]. Overall, restricting the interval between IUS and ileocolonoscopy to 30 days resulted in broadly similar diagnostic performance compared with the overall analyses (Figure 1 and Figure S2).

Although time-specific SICUS-positive rates were not reported, anastomotic BWT > 3.5 mm identified 100% of cases with endoscopic lesions (Rutgeerts i1–i4) in the study by Pallotta et al. (58 patients) [31]. Notably, in six patients with i1 early recurrence 6 months after surgery, all were found to have SICUS BWT between 5 and 10 mm. In four patients with a score of 0 at ileocolonoscopy, CD-related recurrent lesions, confirmed by MRI, were detected at SICUS as BWT ranging between 5 mm and 12 mm.

#### 3.5.3. Colour Doppler

**Figure 5:**
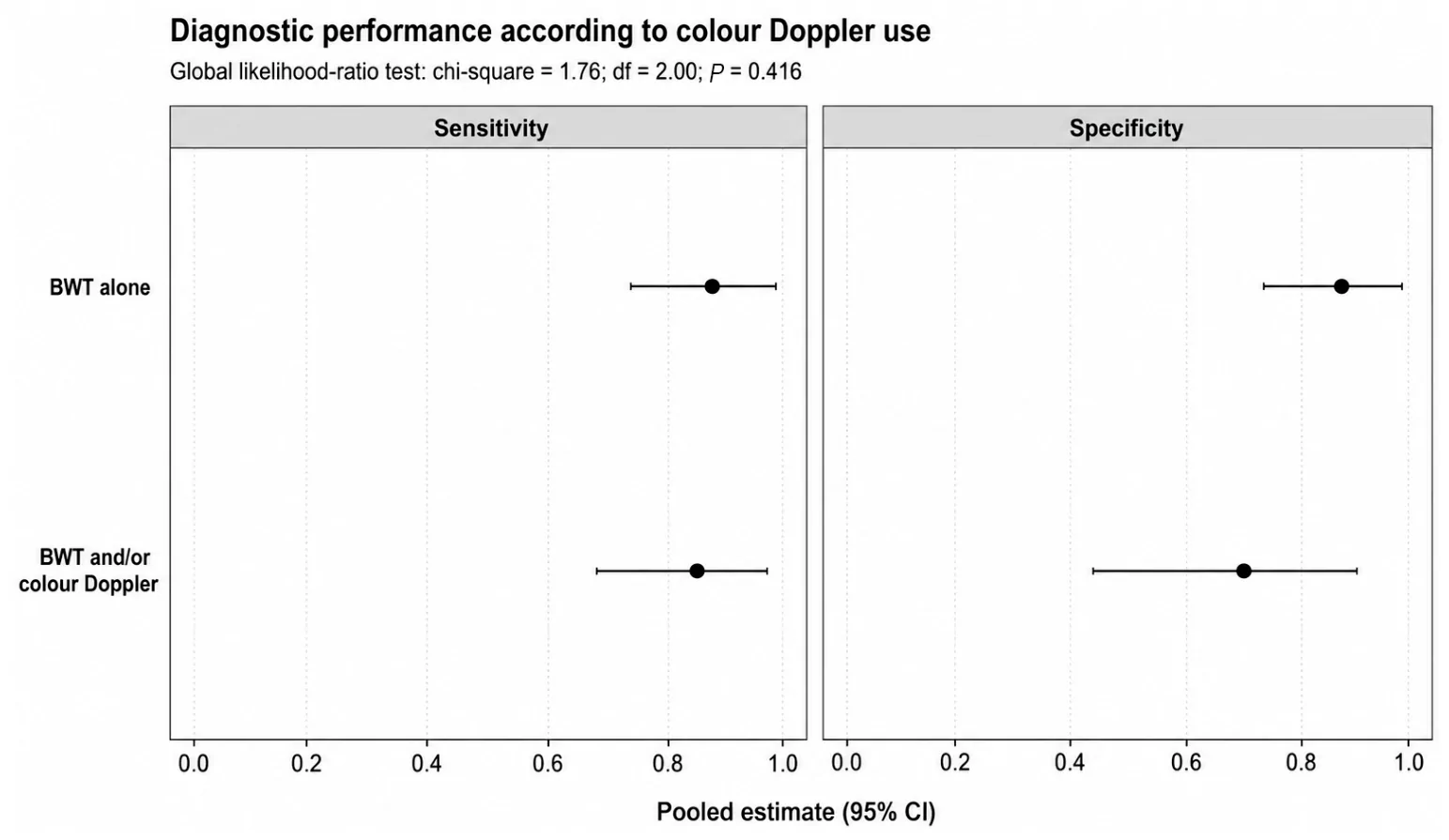
Diagnostic performance of bowel-wall thickness with or without colour Doppler.

In an exploratory subgroup analysis of five conventional IUS studies [26,28,30,33,36], pooled sensitivity was similar between analyses of BWT-only and combined BWT plus colour Doppler: 0.87 (95% CI, 0.76–0.94) versus 0.85 (95% CI, 0.69–0.94), respectively. Specificity was numerically higher for BWT alone but did not reach statistical significance: 0.89 (95% CI, 0.73–0.96) versus 0.72 (95% CI, 0.43–0.90) (χ^2^ = 1.76; df = 2; *p* = 0.416; Figure 5).

#### 3.5.4. Length of Neoterminal Ileal Thickening

Two studies assessed the extent of neoterminal ileal wall thickening in relation to postoperative endoscopic recurrence [29,31]. Calabrese et al. (72 patients) found that lesion extent was greater in patients with severe endoscopic recurrence (Rutgeerts ≥ i3) than in those with conventional (i2) endoscopic recurrence (median, 10 cm vs. 5; *p* < 0.001). In 22 patients with an anastomotic stenosis preventing endoscopic assessment of the proximal neoterminal ileum, SICUS demonstrated a median lesion extent of 13.75 cm. Pallotta et al. (58 patients) similarly reported median intramural lesion extents of 1, 10, 10, and 16 cm for Rutgeerts i1, i2, i3, and i4, respectively, with significantly greater extension for i2–i4 than for i1. Lesion extent and anastomotic BWT were independently associated with Rutgeerts severity, and their combination yielded an AUC of 0.95 for discriminating i0 from i1–i4 and 0.90 for discriminating i0 from i1 [31].

### 3.6. Modified Rutgeerts Classification

Evidence using the modified Rutgeerts score was limited to two studies comprising 52 postoperative patients [35,38]. Allocca et al. identified modified Rutgeerts ≥ i2b recurrence in 6 of 17 patients but did not report diagnostic accuracy estimates [35], whereas Dragoni et al. demonstrated strong correlations between postoperative endoscopic severity according to the modified Rutgeerts classification and all evaluated IUS scores in 35 resected patients [38]. The strongest correlation was observed for the International Bowel Ultrasound Segmental Activity Score (IBUS-SAS; ρ = 0.88), followed by the Bowel Ultrasound Score (BUSS; ρ = 0.84), the Simple Ultrasound Score (Simple-US; ρ = 0.84), and the Simple Ultrasound Score for CD (SUS-CD; ρ = 0.78) [38]. These data could not be included in a meta-analysis.

### 3.7. Pre-Operative IUS and Postoperative Outcomes

Few studies have assessed the role of pre-operative IUS for postoperative risk stratification. In the study by Maconi et al. (85 patients), pre-operative maximum BWT and bowel-wall stratification (BWS) were not associated with recurrence, whereas a greater pre-operative length of bowel-wall thickening was associated with surgical recurrence (28.4 ± 10.7 cm vs. 21.0 ± 11.2 cm; *p* = 0.04) [47]. Parente et al. similarly found no independent prognostic association for pre-operative BWT, BWS pattern, or length of bowel involvement [48].

### 3.8. Intestinal Ultrasound Findings Associated with Future Intestinal Surgery

Seven studies comprising 1060 patients evaluated the association between IUS findings and future intestinal surgery in broader CD populations including newly diagnosed, active, severe or refractory, and established CD populations that were not restricted to postoperative surveillance [40,41,42,43,44,45,46,47,48]. Only one study evaluated primary intestinal resection [42], five included mixed cohorts of patients with and without previous intestinal surgery [40,41,43,44,45], and previous-surgery status was not reported in one study [46]. Data on the role of IUS in predicting the need for re-resections were not identified.

#### 3.8.1. Mixed Primary-Resection and Re-Resection Cohorts

**Figure 6:**
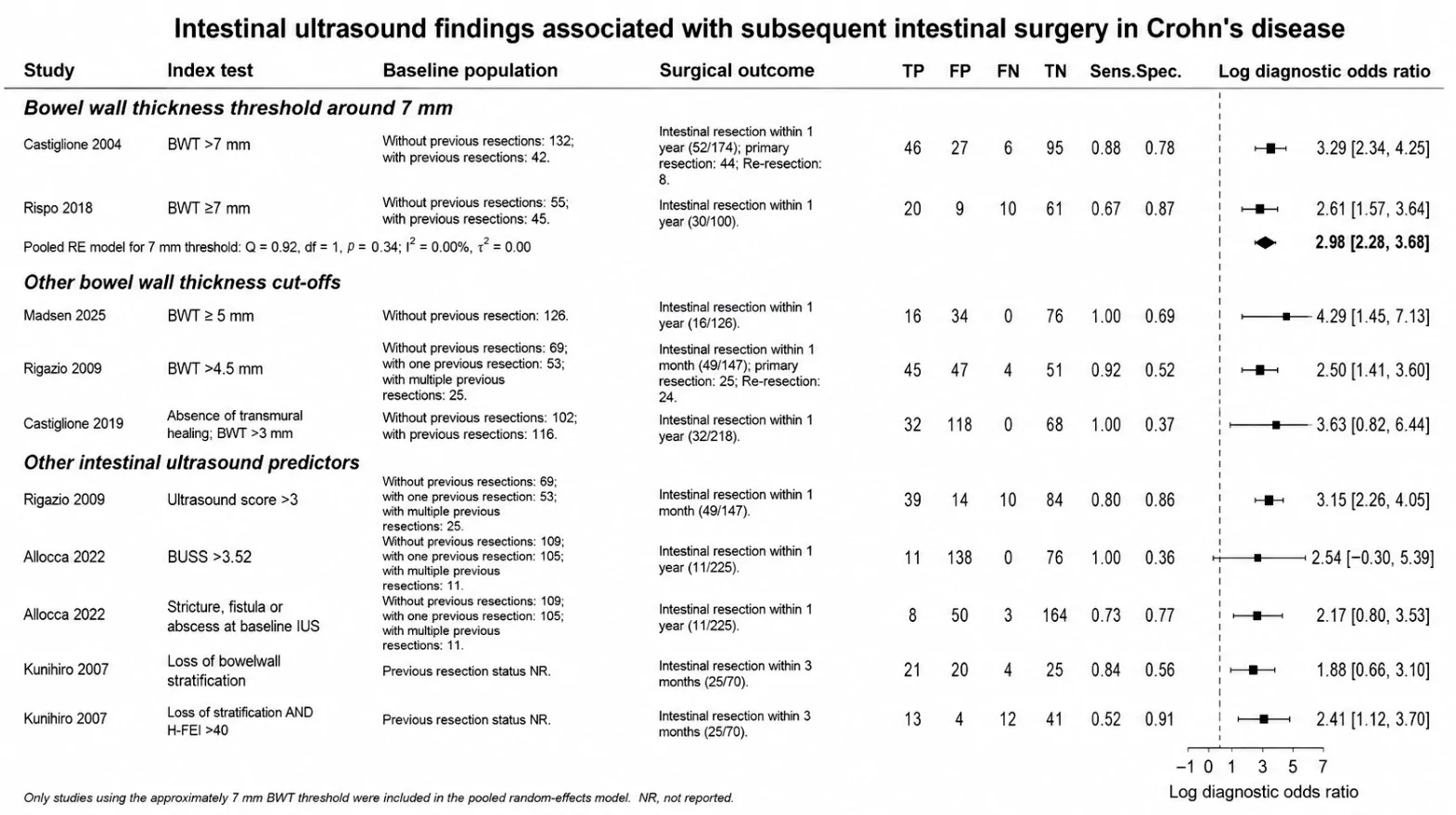
Forest plot of exploratory IUS findings associated with subsequent intestinal surgery.

In an exploratory meta-analysis based on two cohort studies (274 patients) including both primary resections and re-resections, BWT ≥ 7.0 mm was associated with increased risk of intestinal surgery within one year (pooled OR, 19.70; 95% CI, 9.80–39.70; I^2^ = 0%; Figure 6) [40,41]. The studies in this meta-analysis did not report specific risks for primary resections or re-resections. Castiglione et al. included 132 patients without previous intestinal surgery and 42 previously resected patients, with 44 primary resections and eight re-resections occurring during follow-up [40], and Rispo et al. included 55 patients without previous surgery and 45 previously resected patients [41]. In these individual studies, sensitivity ranged from 66.6% to 88.5% and specificity from 78.2% to 87.1% [40,41].

In terms of data not eligible for meta-analysis, all were from mixed cohorts of primary resections and re-resections. Rigazio et al. (147 patients) evaluated 69 patients without previous intestinal resection and 78 previously resected patients [43], of whom 25 patients underwent primary resection, and 24 underwent re-resection [43]; in this overall cohort, a lower BWT threshold (>4.5 mm) achieved higher sensitivity (91.8%) but lower specificity (52.0%). Castiglione et al. (218 patients) also evaluated a cohort in which 116 patients had undergone previous intestinal surgery; however, resections were not classified further [44]. In this study, absence of transmural healing, defined by persistent BWT > 3.0 mm, was associated with a higher frequency of bowel resections within one year [44] (Figure 6). Finally, Alloca et al. included 109 and 116 patients without and with previous resections, respectively [45]; in this study, BUSS > 3.52 and baseline stricture, fistula, or abscesses were associated with a higher frequency of subsequent surgery [45].

In terms of longitudinal changes in BWT, Maconi et al. (85 patients) reported that unchanged or worsening BWT six months after surgery was associated with increased risks of intestinal surgery (aHR, 16.15; 95% CI, 2.87–90.75) [47].

(70 patients) found that loss of bowel-wall stratification independently predicted intestinal surgery within three months (adjusted OR, 5.98; 95% CI, 1.40–25.10), while combining this feature with increased harmonic flash-echo intensity (>40) improved Beyond BWT, Kunihiro et al. specificity to 91.1% [46].

#### 3.8.2. Primary Intestinal Resection

Madsen et al. (126 patients) evaluated primary intestinal resection in a newly diagnosed CD cohort among whom a baseline BWT cut-off of 5.0 mm yielded a sensitivity of 100% and specificity of 69%, while an IBUS-SAS threshold of 63 yielded a sensitivity of 100% and specificity of 73% [42].

#### 3.8.3. Postoperative IUS Predictors of Symptomatic Recurrence

Longitudinal changes in BWT were associated with symptomatic recurrence. Maconi et al. (85 patients) found that unchanged or worsened BWT at 6 months predicted clinical recurrence (aHR, 9.98; 95% CI, 3.48–28.56) [47]. Parente et al. found that unchanged or worsened BWT predicted symptomatic recurrence (aHR, 8.86; 95% CI, 3.37–23.25), while BWT ≥ 6.0 mm independently predicted symptomatic recurrence (aHR, 6.52; 95% CI, 2.75–15.44) [48].

## 4. Discussion

In this systematic review and meta-analysis, IUS demonstrated high overall diagnostic accuracy for postoperative endoscopic recurrence of CD, although its accuracy depended on the endoscopic definition of recurrence. A BWT threshold of >3.0 provided excellent discrimination for detecting any postoperative endoscopic recurrence (Rutgeerts ≥ i1), with an SROC area under the curve exceeding 0.90, balanced sensitivity (85%) and specificity (83%), a DOR of approximately 31, and favourable positive (5.3) and negative (0.19) likelihood ratios. For clinically relevant endoscopic recurrence (Rutgeerts ≥ i2), a similar BWT threshold yielded a more modest sensitivity of 79%, specificity of 67%, DOR of 8.3, and SROC AUC of 0.76. Higher BWT thresholds of approximately 5 mm more accurately identified severe postoperative endoscopic recurrence (Rutgeerts i3–i4), with a sensitivity of 79%, specificity of 81%, and SROC AUC of 0.86. Further, BWT thresholds ≥7.0 mm were associated with future intestinal surgery in two cohorts that included patients with and without previous intestinal surgery. Although OCUS, CEUS, and other advanced IUS techniques showed promising results in selected studies, none consistently improved diagnostic performance compared with conventional transabdominal IUS. Collectively, these findings support IUS as an accurate, non-invasive modality for postoperative surveillance that complements ileocolonoscopy while providing prognostic information and assessment of transmural disease.

The high specificity observed across analyses supports the role of IUS as a surveillance tool, with most patients without endoscopic recurrence correctly classified as test negative. The high DOR and SROC AUC for detecting any postoperative endoscopic recurrence (Rutgeerts ≥ i1) indicate excellent overall discrimination, whereas the LR− below 0.20 suggests that a negative IUS substantially reduces the probability of postoperative endoscopic recurrence. Conversely, LR+ around 5 indicates that a positive IUS meaningfully increases but does not confirm recurrence and therefore supports IUS as a triage tool rather than a replacement for ileocolonoscopy [5,11,12]. Importantly, diagnostic performance for detecting conventional postoperative endoscopic recurrence (Rutgeerts ≥ i2) was more modest. Compared with previous meta-analyses evaluating individual ultrasound parameters [49,50], the present study extends the evidence by applying hierarchical bivariate random-effects models that jointly estimate sensitivity and specificity while accounting for their correlation and between-study heterogeneity. Further, the current analysis is restricted to prospective studies evaluating clinically relevant recurrence definitions, BWT thresholds, ultrasound modality, timing of assessment, and prognostic associations with intestinal surgery, thereby providing a comprehensive framework for postoperative IUS surveillance.

Other non-invasive surrogate markers for postoperative monitoring of CD include C-reactive protein (CRP) and fecal calprotectin. A recent meta-analysis reported poor sensitivity (0.45 (95% CI, 0.33–0.58)) but relatively high specificity for CRP (0.83 (95% CI, 0.69–0.92)), whereas fecal calprotectin demonstrated higher sensitivity (0.76 (95% CI, 0.70–0.82)) at the expense of lower specificity (0.66 (95% CI, 0.56–0.75)) [51]. Recently, the American Gastroenterological Association conditionally recommended in favour of a strategy that uses fecal calprotectin-based monitoring, using a cutoff of <50 mg/g, over routine endoscopic evaluation to rule out postoperative endoscopic recurrence in asymptomatic patients with CD after surgically induced remission within the past 12 months and who are either receiving postoperative pharmacologic prophylaxis or at low baseline risk of postoperative endoscopic recurrence (regardless of postoperative prophylaxis) [52]. Based on the current study, we propose a strategy combining fecal calprotectin with IUS for this patient population, providing complementary information on both luminal inflammatory activity and transmural and structural changes [25,39].

The timing of postoperative IUS is another important consideration. When IUS is performed after bowel preparation, bowel cleansing and luminal distension may influence bowel-wall appearance and BWT, potentially introducing measurement bias. Most included studies performed IUS and ileocolonoscopy concurrently, although at varying intervals after surgery. These studies primarily assess the cross-sectional diagnostic accuracy of IUS rather than its ability to predict future recurrence [25]. In exploratory meta-regression, no statistically significant association was detected between diagnostic performance and the interval from surgery to IUS; however, the limited number of contributing studies restricts statistical power [25]. In contrast, longer time between IUS and endoscopy was associated with lower specificity. Accordingly, a negative early postoperative IUS at 3 months is not sufficient to replace guideline-recommended endoscopic surveillance [53,54]. Instead, the findings of this systematic review suggest a role for serial IUS examinations and longitudinal changes in ultrasound parameters in postoperative prognostic assessment [44,47,48].

Beyond diagnosis, longitudinal changes in BWT may provide clinically meaningful prognostic information. Persistent or progressive bowel-wall thickening was consistently associated with clinical recurrence and intestinal surgery [47,48], whereas early reductions in BWT and achievement of transmural healing were associated with favourable outcomes [42,44,47,48]. These findings suggest that postoperative IUS may serve not only as a diagnostic tool but also as a dynamic biomarker of postoperative disease evolution and treatment response. Taken together, these findings suggest an association between IUS-detected transmural abnormalities and subsequent clinical and surgical outcomes. However, the available studies were not designed or sufficiently adjusted to determine whether this association is independent of mucosal recurrence, disease phenotype, treatment, or other relevant confounders. Importantly, the need for surgery in CD is associated with numerous factors, including stricturing or penetrating phenotype, disease extent, treatment response, previous surgery, and clinical decision-making. Future interventional studies should therefore determine the role of serial IUS in the management of postoperative CD and determine whether treatment strategies guided by serial ultrasound assessments and transmural remission as a formal treatment target improve long-term postoperative outcomes [55].

The available evidence also provides important insights into the role of advanced ultrasound techniques and additional sonographic parameters. Although OCUS and CEUS showed promising diagnostic performance in selected studies, neither technique consistently outperformed conventional transabdominal IUS. The apparently high sensitivity of OCUS was largely driven by studies with a high prevalence of recurrence, resulting in imprecise specificity estimates [27]. Likewise, CEUS and composite ultrasound scores appeared particularly useful for identifying severe recurrence but were evaluated using heterogeneous protocols and predominantly single-centre cohorts without external validation [33,36,37,38]. Beyond BWT, potentially relevant ultrasound features include colour Doppler signal, reflecting mural vascularity [30,33,35,36,37,38,39], mesenteric lymph nodes [35,39], and composite ultrasound scores [33,36,37,38]. In the current study, explorative meta-regression incorporating both BWT and colour Doppler did not significantly improve pooled diagnostic performance compared with BWT alone. Although sensitivity was comparable and specificity appeared lower for Doppler-inclusive definitions, this finding should be interpreted cautiously because only two studies evaluating colour Doppler contributed to the pooled analyses [30,33]. These findings support BWT as the cornerstone of postoperative IUS assessment, whereas advanced ultrasound techniques and additional sonographic parameters should currently be regarded as complementary tools requiring further prospective validation before routine implementation.

Interpretation of the available evidence is further influenced by differences in the endoscopic reference standard. Most studies used the original Rutgeerts classification, limiting extrapolation to the modified Rutgeerts ≥ i2b definition, which has been associated with a higher risk of clinical and surgical recurrence [56], and which has been evaluated in only two IUS studies. Consequently, the diagnostic performance of IUS for modified Rutgeerts ≥ i2b remains uncertain and warrants prospective evaluation. Nevertheless, studies have questioned the clinical significance of discriminating between i2a and i2b [57,58], reinforcing the clinical relevance of our study findings.

Strengths of this systematic review and meta-analysis include the restriction to prospective studies, the application of hierarchical bivariate random-effects models that jointly estimate sensitivity and specificity while accounting for their correlation and between-study heterogeneity, and the separate synthesis of diagnostic accuracy according to specific BWT thresholds and clinically relevant definitions of postoperative endoscopic recurrence. Furthermore, by integrating both diagnostic and prognostic evidence, this review provides a comprehensive assessment of IUS across the postoperative disease course. These strengths should be interpreted alongside several limitations. First, the evidence base was predominantly derived from single-centre cohorts involving highly experienced sonographers, which may limit generalizability. Considerable heterogeneity in ultrasound techniques, endoscopic reference standards, BWT thresholds, additional sonographic parameters, and timing of assessments further limited the number of studies contributing to pooled analyses. Second, both the analysis of BWT ≥ 7 mm in relation to subsequent intestinal surgery and the pooled analysis of conventional postoperative endoscopic recurrence (Rutgeerts ≥ i2) were based on only two studies and should therefore be considered exploratory. Importantly, recent evidence has highlighted the potential for misclassification of postoperative endoscopic recurrence and the importance of accounting for anastomotic configuration when interpreting endoscopic findings; however, these factors were not addressed in the available literature [53,58]. Finally, evidence supporting OCUS, CEUS, and the modified Rutgeerts classification remains limited [59]. Future prospective studies should include centrally read IUS parameters and calprotectin for early successive monitoring and prediction of endoscopic recurrence.

## 5. Conclusions

In conclusion, IUS is an accurate, non-invasive, and repeatable modality for postoperative monitoring of CD demonstrating high diagnostic performance for endoscopic recurrence with balanced sensitivity and specificity, favourable likelihood ratios, and good overall discriminatory ability. However, diagnostic performance varies according to the definition and severity of recurrence and is more modest for conventional postoperative endoscopic recurrence (Rutgeerts ≥ i2) than for any postoperative endoscopic recurrence (Rutgeerts ≥ i1). Evidence evaluating IUS against the modified Rutgeerts classification was limited; consequently, the accuracy of IUS for modified Rutgeerts ≥ i2b and for distinguishing anastomotic from neoterminal ileal lesions remains uncertain. While ileocolonoscopy remains the reference standard, serial IUS provides a complementary, non-invasive approach for repeated assessment of transmural disease activity and prognostic risk stratification and may help inform the timing of endoscopic reassessment.

## Figures and Tables

**Table 1:** Characteristics of prospective studies evaluating postoperative endoscopic recurrence.

Abbreviations: Study, Year (Ref.)	N Centres	Patients, n	Type of IUS	Time from Surgery to IUS	Time Between IUS and Endoscopy	Evaluated IUS Parameters and Cut-Offs	Reference Standard and Outcome Definition	Study Findings
DiCandio et al. 1986 [23]	1	32	Conventional IUS	Mean 4.5 years (range, 1–12)	≤2 weeks	Ileocolonic BWT > 5.0 mm; wall architecture; complications	Contrast radiography plus endoscopy/biopsy; postoperative relapse; no Rutgeerts classification	Se 0.82; Sp 1.00; accuracy 0.94
Andreoli et al. 1998 [24]	1	41	Conventional IUS	Mean 35.4 months (range, 3–105)	≤14 days	Neoterminal-ileal BWT > 5.0 mm	Ileocolonoscopy; typical lesions in the neoterminal ileum and/or ICA; Rutgeerts-derived mild, moderate, and severe grading	Se 0.81; Sp 0.86; accuracy 0.83
Orlando et al. 2006 [25]	2	50	Conventional IUS	3 months	9-month forward-prediction interval; endoscopy at 12 mo	BWT ≥ 5.0 mm	Ileocolonoscopy at 12 months; Rutgeerts ≥ i2, defined as ≥5 aphthous lesions	Se 0.26; Sp 0.90
Rispo et al. 2006 [26]	1	45	Conventional IUS	12 months	≤7 days	Neoterminal-ileal/ICA BWT > 3.0 mm; BWT > 5.0 mm for severe recurrence	Ileocolonoscopy; original Rutgeerts ≥ i1; severe recurrence i3–i4	Any recurrence: Se 0.79, Sp 0.95; severe recurrence: Se 0.94, Sp 1.00
Biancone et al. 2007 [27]	1	22	Oral-contrast IUS	12 months; subgroup assessed at 3, 6, and 12 months	Sequential same-visit assessment at 12 months	Perianastomotic/neoterminal-ileal BWT > 3.0 mm; stiff loop; dilatation > 2.5 cm; stricture < 1 cm; fistula; abscess	Ileocolonoscopy; original Rutgeerts ≥ i1; secondary analysis restricted to the neoterminal ileum	Se 1.00; Sp 0.00; IUS was positive in 4/10, 8/10, and 10/10 patients at 3, 6, and 12 mo, respectively
Castiglione et al. 2008 [28]	1	40	Conventional IUS and oral-contrast IUS	12 months	≤7 days	ICA/neoterminal-ileal BWT ≥ 3.0 mm; severe recurrence: conventional-IUS BWT ≥ 5.0 mm or oral-contrast-IUS BWT ≥ 4.0 mm	Ileocolonoscopy; original Rutgeerts ≥ i1; severe recurrence i3–i4	Conventional IUS: Se 0.77, Sp 0.94; oral-contrast IUS: Se 0.82, Sp 0.94. Severe recurrence: conventional IUS: Se 0.93, Sp 0.96; oral-contrast IUS: Se 0.86, Sp 0.96
Calabrese et al. 2009 [29]	1	72	Oral-contrast IUS	Median 18 months (range, 3–396)	≤6 months; clinically active subgroup median 1 month	Perianastomotic BWT > 3.0 mm for ≥4 cm; dilatation > 2.5 cm; stricture < 1 cm; fistula; abscess	Ileocolonoscopy; original Rutgeerts ≥ i1; ordinal analysis of recurrence severity	Se 0.93; Sp 0.20; accuracy 0.88; BWT correlated with Rutgeerts score (r = 0.67)
Onali et al. 2010 [32]	1	25 at 1 y; 21 at 2 y; 15 at 3 y	Oral-contrast IUS	12, 24, and 36 months	At 12 months, ileocolonoscopy followed by IUS; exact interval NR	BWT > 3.0 mm; stiff loop; dilatation > 2.5 cm; stricture < 1 cm; fistula; abscess	Ileocolonoscopy at 1 and 3 years; original Rutgeerts ≥ i1; secondary ≥ i2 analysis	At 1 y: Se 1.00, Sp 0.00; 1-y BWT did not predict clinical recurrence at 2 y (*p* = 0.19)
Pallotta et al. 2010 [31]	1	58	Oral-contrast IUS	6 months, 12 months, and every 6–12 months thereafter (range, 6–100 months)	≤2 weeks; different days and random order	ICA BWT > 3.5 mm; neoterminal-ileal BWT > 3.0 mm and length of thickening; stenosis; dilatation	Ileocolonoscopy; original Rutgeerts 0 versus 1–4 and ordinal categories 0, 1, and ≥i2	ICA BWT > 3.5 mm detected all endoscopic lesions; ICA BWT combined with lesion length yielded AUC 0.95
Paredes et al. 2010 [30]	1	33	Conventional IUS and colour Doppler	Mean 87.7 months (SD 75.4)	≤3 days	Neoterminal-ileal BWT > 3.0 mm and/or positive colour Doppler; moderate/severe recurrence: BWT > 5.0 mm and/or Doppler grade 2–3	Ileocolonoscopy; original Rutgeerts ≥ i1; moderate/severe recurrence i3–i4	Any recurrence: Se 0.77, Sp 0.57; moderate/severe recurrence: Se 0.80, Sp 0.78
Paredes et al. 2013 [33]	1	60	Conventional IUS, colour Doppler and CEUS	60 mo (SD 71)	≤3 days	Neoterminal-ileal BWT > 3.0 mm and/or positive colour Doppler; score 2: BWT > 5.0 mm or BWCE > 46%; score 3: BWT > 5.0 mm or BWCE > 70% or fistula	Ileocolonoscopy; original Rutgeerts ≥ i1; moderate/severe recurrence i3–i4	Conventional IUS: Se 0.90, Sp 0.82; score 2: Se 0.98, Sp 1.00; score 3: Se 0.94, Sp 0.73
Onali et al. 2016 [34]	1	40	Oral-contrast IUS	12 months	NR	Perianastomotic/neoterminal-ileal BWT ≥ 3.0 mm	Ileocolonoscopy; original Rutgeerts ≥ i1 and secondary ≥ i2 analysis; clinical recurrence assessed through 5 years	IUS and endoscopy each identified recurrence in 39/40 patients; 1-y IUS did not discriminate clinical recurrence
Allocca et al. 2018 [35]	1	60	Conventional IUS and Power Doppler	NR	≤3 days	BWT > 3.0 mm; BWP; BWF; ulcers; strictures; fistulas; abscesses; lymph nodes; mesenteric hypertrophy	Ileocolonoscopy; modified Rutgeerts ≥ i2b in the postoperative subgroup	Recurrence occurred in 6/17 postoperative patients; postoperative-specific Se and Sp were NR
Martínez et al. 2019 [36]	1	108	Conventional IUS, colour Doppler and CEUS	Mean 6 years (range, 3 months–30 years)	Median 1 month 28 days (range, 0–86 days)	ICA/neoterminal-ileal BWT ≥ 3.0 mm; BWCE ≥ 46%; severe recurrence BWT ≥ 5.0 mm; severe model: BWT ≥ 6.0 mm or BWT 5–6 mm plus BWCE ≥ 70% or complications	Ileocolonoscopy; original Rutgeerts ≥ i1; severe recurrence i3–i4	BWT ≥ 3.0 mm: accuracy 0.91; BWT plus BWCE: Se 0.91, Sp 0.89; severe model: Se 0.90, Sp 0.87
Macedo et al. 2022 [37]	1	39	Conventional IUS and Power Doppler	Median 9 years (IQR, 3–12)	Same day; IUS performed first	BWT at the thickest ileocolic segment; abnormal IUS: BWT > 3.0 mm and/or Limberg > 1; ROC-derived BWT ≥ 3.9 mm	Ileocolonoscopy; original Rutgeerts ≥ i2	Composite IUS: Se 0.89, Sp 0.62; BWT ≥ 3.9 mm: Se 0.89, Sp 0.71; IUS severity AUC 0.82
Dragoni et al. 2023 [38]	1	73	Conventional IUS and colour Doppler	NR	Median 3 weeks (IQR, 2–5); maximum 6 weeks	IBUS-SAS; BUSS; Simple-US; SUS-CD; BWT; CDS; BWS; i-fat	Ileocolonoscopy; modified Rutgeerts ≥ i2b; severe recurrence i4	Correlations with Rutgeerts score were ρ = 0.88 for IBUS-SAS, 0.84 for BUSS, 0.84 for Simple-US, and 0.78 for SUS-CD
Furfaro et al. 2023 [39]	3	91	Conventional IUS and colour Doppler	Within 1 y; exact IUS timing NR; endoscopy median 6.5 months (range, 4–12)	≤90 days	Neoterminal-ileal/ICA BWT ≥ 3.0 mm; mesenteric lymph nodes; FC ≥ 50 μg/g	Ileocolonoscopy; original Rutgeerts ≥ i2	BWT ≥ 3.0 mm: Se 0.77, Sp 0.65; BWT plus FC using an AND rule: Se 0.65, Sp 0.93; lymph nodes: Se 0.35, Sp 0.97; BWT per 1 mm increase: aOR 2.43

aHR, adjusted hazard ratio; aOR, adjusted odds ratio; AUC, area under the receiver operating characteristic curve; BUSS, Bowel Ultrasound Score; BWCE, bowel-wall contrast enhancement; BWF, bowel-wall flow; BWP, bowel-wall pattern; BWS, bowel-wall stratification; BWT, bowel-wall thickness; CDS, colour Doppler signal; CEUS, contrast-enhanced ultrasonography; FC, fecal calprotectin; H-FEI, harmonic flash-echo imaging; ICA, ileocolonic anastomosis; IBUS-SAS, International Bowel Ultrasound Segmental Activity Score; i-fat, inflammatory mesenteric fat; IQR, interquartile range; IUS, intestinal ultrasonography; N/A, not applicable; NR, not reported; ROC, receiver operating characteristic; SD, standard deviation; Se, sensitivity; Simple-US, Simple Ultrasound Score; Sp, specificity; SUS-CD, Simple Ultrasound Score for Crohn’s Disease.

**Table 2:** Characteristics of prospective studies evaluating prognostic outcomes of IUS in CD.

Abbreviations: Study, Year (Ref.)	N Centres	Patients, n	Type of IUS	Time from Surgery to IUS	Time Between IUS and Endoscopy	Evaluated IUS Parameters and Cut-Offs	Reference Standard and Outcome Definition	Study Findings
**Panel A: Postoperative longitudinal prognostic studies**								
Maconi et al. 2001 [47]	1	85	Conventional IUS	Pre-operative and 6 months postoperative, then every 6 months; median follow-up 28.4 months (range 3–70)	N/A	maximum BWT; length of thickening; wall pattern; BWT ≥ 4.0 mm	Clinical recurrence requiring medium- or high-dose corticosteroids; surgical recurrence requiring a new procedure	Unchanged/worsened 6-mo BWT predicted clinical recurrence (aHR 9.98) and surgical recurrence (aHR 16.15); greater pre-operative disease length was associated with surgery
Parente et al. 2004 [48]	1	127	Conventional IUS	Pre-Operative, 6 months, 12 months, and annually thereafter, median follow-up 41.0 months	N/A	BWT ≥ 4.0 mm; 12-mo BWT > 6.0 mm	Symptomatic recurrence requiring systemic corticosteroids	Unchanged/worsened 12-mo BWT predicted symptomatic recurrence (aHR 8.90); BWT > 6.0 mm was also predictive (aHR 6.50)
**Panel B: Studies evaluating future intestinal surgery in broader Crohn’s disease populations**								
Castiglione et al. 2004 [40]	1	174	Conventional IUS	NR	N/A	BWT > 7.0 mm	Intestinal resection within 12 months	BWT > 7.0 mm: Se 0.88; Sp 0.78; AUC 0.83; aOR 19.52
Kunihiro et al. 2007 [46]	1	70	Conventional IUS and H-FEI	N/A	N/A	BWT ≥ 4.0 mm; loss of BWS; H-FEI echo intensity > 40	Bowel resection within 3 months	Loss of BWS: aOR 5.98; H-FEI echo intensity: OR 1.02 per unit; combined rule: Se 0.52, Sp 0.91
Rigazio et al. 2009 [43]	1	147	Conventional IUS	N/A	N/A	BWT > 4.5 mm; disrupted BWS; fistula/abscess; stenosis; composite IUS score	Actual intestinal surgery within 30 days; controls remained surgery-free for ≥1 year	BWT > 4.5 mm: Se 0.92, Sp 0.52, OR 12.21; disrupted BWS: OR 16.24; composite score correctly classified 84% of patients
Rispo et al. 2018 [41]	1	100	Conventional IUS	N/A	≤1 week	BWT ≥ 7.0 mm; small-bowel disease extent ≥ 33 cm; complications	Major intestinal surgery within 12 months; minor and perianal procedures excluded	BWT ≥ 7.0 mm: Se 0.67, Sp 0.87, AUC 0.87, aOR 15.80; risk-matrix probabilities ranged from 0.48% to 87.5%
Castiglione et al. 2019 [44]	1	218	Conventional IUS	N/A; baseline after 2 y of anti-TNF therapy	≤1 week	Transmural healing: BWT ≤ 3.0 mm in all affected segments; mucosal healing only; no healing	CD-related surgery within 12 months; major intestinal resection/colectomy and minor procedures included	Surgery occurred in 0%, 10.0%, and 35.5% of patients with transmural, mucosal-only, and no healing, respectively; HR 0.94 versus mucosal healing
Allocca et al. 2022 [45]	1	225	Conventional IUS and Power Doppler	N/A; baseline before 12-mo follow-up	3 days–3 months	BWT ≤ 3.0 mm; BWF; BWP; complications; BUSS > 3.52	Individual need for surgery within 12 mo; composite adverse disease course also assessed	Surgery occurred in 13.7% with baseline complications versus 1.7% without; complications independently predicted surgery (aOR 7.56); BUSS was not independently predictive of surgery
Madsen et al. 2025 [42]	2	201	Conventional IUS and colour Doppler	N/A; baseline at diagnosis, median 5 days after diagnosis (IQR, 0–22)	NR	Terminal-ileal IBUS-SAS; BWT; CDS; BWS; i-fat; cut-offs: IBUS-SAS 63 and BWT 5.0 mm	Ileocecal resection within 12 months of diagnosis	IBUS-SAS 63: AUC 0.92, Se 1.00, Sp 0.73; BWT 5.0 mm: AUC 0.87, Se 1.00, Sp 0.69

aHR, adjusted hazard ratio; aOR, adjusted odds ratio; AUC, area under the receiver operating characteristic curve; BUSS, Bowel Ultrasound Score; BWCE, bowel-wall contrast enhancement; BWF, bowel-wall flow; BWP, bowel-wall pattern; BWS, bowel-wall stratification; BWT, bowel-wall thickness; CDS, colour Doppler signal; CEUS, contrast-enhanced ultrasonography; FC, faecal calprotectin; H-FEI, harmonic flash-echo imaging; ICA, ileocolonic anastomosis; IBUS-SAS, International Bowel Ultrasound Segmental Activity Score; i-fat, inflammatory mesenteric fat; IQR, interquartile range; IUS, intestinal ultrasonography; N/A, not applicable; NR, not reported; ROC, receiver operating characteristic; SD, standard deviation; Se, sensitivity; Simple-US, Simple Ultrasound Score; Sp, specificity; SUS-CD, Simple Ultrasound Score for Crohn’s Disease.

**Table 3:** Pooled diagnostic accuracy of intestinal ultrasonography for postoperative endoscopic recurrence.

Endoscopic Outcome and Index-Test Group	Studies, n	Patients, n	TP	FN	FP	TN	Bivariate Sensitivity, 95% CI	Bivariate Specificity, 95% CI	LR+, 95% CI	LR−, 95% CI	DOR, 95% CI	AUC, 95% CI	Heterogeneity
Any postoperative endoscopic recurrence (original Rutgeerts ≥ i1); conventional transabdominal IUS with study-defined BWT > 3.0 mm, with or without colour Doppler	5	286	185	26	12	63	0.85 (0.75–0.92)	0.83 (0.65–0.92)	5.27 (2.5010.50)	0.19 (0.10–0.30)	30.80 (10.60–70.70)	0.91 (0.75–0.93)	I^2^ = 0.00%; τ^2^ = 0.00
Conventional postoperative endoscopic recurrence (original Rutgeerts ≥ i2); conventional transabdominal IUS with study-defined BWT ≥ 3.0 or ≥ 3.9 mm	2	130	62	16	17	35	0.79 (0.68–0.87)	0.67 (0.53–0.79)	2.47 (1.64–3.71)	0.33 (0.19–0.50)	8.31 (3.42–17.00)	0.76 (0.76–0.78)	I^2^ = 29.46%; τ^2^ = 0.22
Severe postoperative endoscopic recurrence (original Rutgeerts i3–i4); conventional transabdominal IUS with study-defined BWT > 5.0 or ≥ 5.0 mm	4	246	101	26	18	101	0.79 (0.70–0.85)	0.81 (0.72–0.88)	4.29 (2.79–6.49)	0.27 (0.19–0.37)	16.80 (8.12–30.90)	0.86 (0.80–0.94)	I^2^ = 79.21%; τ^2^ = 5.71
Any postoperative endoscopic recurrence (original Rutgeerts ≥ i1); oral-contrast ultrasonography with study-defined BWT around 3.0 mm, with or without ancillary findings	5	199	164	9	7	19	0.93 (0.84–0.97)	0.52 (0.14–0.87)	2.43 (1.10–6.89)	0.17 (0.07–0.42)	18.70 (2.75–66.10)	0.91 (0.52–0.95)	I^2^ = 72.46%; τ^2^ = 3.72
BWT ≥ 7.0 mm and intestinal surgery within one year	2	274	66	16	36	156					19.70 (9.80–39.70)		I^2^ = 0.00%; τ^2^ = 0.00

TP, true positive; FN, false negative; FP, false positive; TN, true negative; CI, confidence interval; LR+, positive likelihood ratio; LR-, negative likelihood ratio; DOR, diagnostic odds ratio; AUC, area under the curve; IUS, intestinal ultrasonography; BWT, bowel-wall thickness.

## Data Availability

Data are presented in the current manuscript and its Supplementary Materials. Extracted study-level dataset and supplementary analytical data will be made available upon reasonable request to the corresponding author.

## Supplementary Materials

The following supporting information can be downloaded at: https://www.mdpi.com/article/10.3390/jcm15187024/s1, Figure S1: Flow chart; Figure S2: Sensitivity analysis with up to 30 days between IUS and endoscopy; Table S1: PRISMA-DTA Checklist; Table S2: Systematic search; Table S3: Intestinal ultrasonography protocols; Table S4: Assessment of risk of biases; Table S5: Bias in included studies of prognostic IUS.

## Abbreviations

The following abbreviations are used in this manuscript:

aHR	adjusted hazard ratio
anti-TNF	Anti-tumour necrosis factor
aOR	adjusted odds ratio
AUC	area under the curve
AUROC	area under the receiver operating characteristic curve
B-mode	brightness mode
BUSS	Bowel Ultrasound Score
BWCE	bowel-wall contrast enhancement
BWF	bowel-wall flow
BWP	bowel-wall pattern
BWS	bowel-wall stratification
BWT	bowel-wall thickness
CD	Crohn’s disease
CDS	colour Doppler signal
CEUS	contrast-enhanced ultrasonography
CI	confidence interval
CRP	C-reactive protein
df	degrees of freedom
DOR	diagnostic odds ratio
FC	fecal calprotectin
FN	false negative
FP	false positive
FPR	false-positive rate
H-FEI	harmonic flash-echo imaging
HR	hazard ratio
IBUS-SAS	International Bowel Ultrasound Segmental Activity Score
ICA	ileocolonic anastomosis
i-fat	inflammatory mesenteric fat
IQR	interquartile range
IUS	intestinal ultrasonography
LR+	positive likelihood ratio
LR−	negative likelihood ratio
mo	months
MRI	magnetic resonance imaging
N/n	number
N/A	not applicable
NR	not reported
OCBS	oral-contrast bowel sonography
OCUS	oral-contrast ultrasonography
OR	odds ratio
POCER	Postoperative Crohn’s Endoscopic Recurrence trial
PRISMA-DTA	Preferred Reporting Items for Systematic Reviews and Meta-Analyses of Diagnostic Test Accuracy Studies
PROSPERO	International Prospective Register of Systematic Reviews
QUIPS	Quality In Prognosis Studies
QUADAS-2	Quality Assessment of Diagnostic Accuracy Studies 2
RE	random-effects
ROC	receiver operating characteristic
SD	standard deviation
Se	sensitivity
SICUS	small intestine contrast ultrasonography
Simple-US	Simple Ultrasound Score
Sp	specificity
SROC	summary receiver operating characteristic
SUS-CD	Simple Ultrasound Score for Crohn’s Disease
TA-IUS	transabdominal intestinal ultrasonography
TN	true negative
TP	true positive
y	years
