# Supplementary material for: Intestinal Ultrasonography for Predicting Postoperative Endoscopic Recurrence and Assessing Risk of Intestinal Surgery in Crohn’s Disease—A Systematic Review and Meta-Analysis of Prospective Studies
Source: J Clin Med. 2026 Sep 10;15(18):7024. doi: 10.3390/jcm15187024 (PMC13607291; doi:10.3390/jcm15187024)

Supplementary Material

Contents

Supplementary Material 1

Supplementary Tables 2

Supplementary Table S1: PRISMA-DTA Checklist 2

Supplementary Table S2: Systematic search 5

Supplementary Table S3: Intestinal ultrasonography protocols 6

Supplementary Table S4: Assessment of risk of biases 9

Supplementary Table S5: Bias in included studies of prognostic IUS 11

References 12

Supplementary Figures 15

Supplementary Figure S1: Flow chart 15

Supplementary Figure S2: Sensitivity analysis with up to 30 days between IUS and endoscopy 16

Supplementary Tables

Supplementary Table S1: PRISMA-DTA Checklist

Section/topic	#	PRISMA-DTA Checklist Item	Reported on page #
**TITLE / ABSTRACT**			
Title	1	Identify the report as a systematic review (+/- meta-analysis) of diagnostic test accuracy (DTA) studies.	1
Abstract	2	Abstract: See PRISMA-DTA for abstracts.	1-2
**INTRODUCTION**			
Rationale	3	Describe the rationale for the review in the context of what is already known.	2
Clinical role of index test	D1	State the scientific and clinical background, including the intended use and clinical role of the index test, and if applicable, the rationale for minimally acceptable test accuracy (or minimum difference in accuracy for comparative design).	2
Objectives	4	Provide an explicit statement of question(s) being addressed in terms of participants, index test(s), and target condition(s).	2
**METHODS**			
Protocol and registration	5	Indicate if a review protocol exists, if and where it can be accessed (e.g., Web address), and, if available, provide registration information including registration number.	2
Eligibility criteria	6	Specify study characteristics (participants, setting, index test(s), reference standard(s), target condition(s), and study design) and report characteristics (e.g., years considered, language, publication status) used as criteria for eligibility, giving rationale.	3
Information sources	7	Describe all information sources (e.g., databases with dates of coverage, contact with study authors to identify additional studies) in the search and date last searched.	2-3
Search	8	Present full search strategies for all electronic databases and other sources searched, including any limits used, such that they could be repeated.	2-3; Supplementary Table S2
Study selection	9	State the process for selecting studies (i.e., screening, eligibility, included in systematic review, and, if applicable, included in the meta-analysis).	3

Data collection process	10	Describe method of data extraction from reports (e.g., piloted forms, independently, in duplicate) and any processes for obtaining and confirming data from investigators.	3-4
Definitions for data extraction	11	Provide definitions used in data extraction and classifications of target condition(s), index test(s), reference standard(s) and other characteristics (e.g. study design, clinical setting).	3-4
Risk of bias and applicability	12	Describe methods used for assessing risk of bias in individual studies and concerns regarding the applicability to the review question.	4
Diagnostic accuracy measures	13	State the principal diagnostic accuracy measure(s) reported (e.g. sensitivity, specificity) and state the unit of assessment (e.g. per-patient, per-lesion).	4
Synthesis of results	14	Describe methods of handling data, combining results of studies and describing variability between studies. This could include, but is not limited to: a) handling of multiple definitions of target condition. b) handling of multiple thresholds of test positivity, c) handling multiple index test readers, d) handling of indeterminate test results, e) grouping and comparing tests, f) handling of different reference standards	4

Page 1 of 2

Section/topic	#	PRISMA-DTA Checklist Item	Reported on page #
Meta-analysis	D2	Report the statistical methods used for meta-analyses, if performed.	4
Additional analyses	16	Describe methods of additional analyses (e.g., sensitivity or subgroup analyses, meta-regression), if done, indicating which were pre-specified.	4
**RESULTS**			
Study selection	17	Provide numbers of studies screened, assessed for eligibility, included in the review (and included in meta-analysis, if applicable) with reasons for exclusions at each stage, ideally with a flow diagram.	4
Study characteristics	18	For each included study provide citations and present key characteristics including: a) participant characteristics (presentation, prior testing), b) clinical setting, c) study design, d) target condition definition, e) index test, f) reference standard, g) sample size, h) funding sources	4-11
Risk of bias and applicability	19	Present evaluation of risk of bias and concerns regarding applicability for each study.	9-14
Results of individual studies	20	For each analysis in each study (e.g. unique combination of index test, reference standard, and positivity threshold) report 2x2 data (TP, FP, FN, TN) with estimates of diagnostic accuracy and confidence intervals, ideally with a forest or receiver operator characteristic (ROC) plot.	6-25
Synthesis of results	21	Describe test accuracy, including variability; if meta-analysis was done, include results and confidence intervals.	12-25

Additional analysis	23	Give results of additional analyses, if done (e.g., sensitivity or subgroup analyses, meta-regression; analysis of index test: failure rates, proportion of inconclusive results, adverse events).	17-25
**DISCUSSION**			
Summary of evidence	24	Summarize the main findings including the strength of evidence.	26-28
Limitations	25	Discuss limitations from included studies (e.g. risk of bias and concerns regarding applicability) and from the review process (e.g. incomplete retrieval of identified research).	27
Conclusions	26	Provide a general interpretation of the results in the context of other evidence. Discuss implications for future research and clinical practice (e.g. the intended use and clinical role of the index test).	28
**FUNDING**			
Funding	27	For the systematic review, describe the sources of funding and other support and the role of the funders.	29

Supplementary Table S2: Systematic search

Per July 1st 2026.

Pubmed	Search	Number of studies in Pubmed	Number of studies in EMBASE
**Search 1**	("Crohn Disease"[Mesh] OR "Crohn Disease"[tiab] OR "Crohn*"[tiab] OR "morbus crohn"[tiab] OR "Inflammatory Bowel Diseases"[Mesh] OR "inflammatory bowel disease"[tiab] OR "IBD"[tiab])	**157,732**	**139,903**
**Search 2**	("Ultrasonography"[Mesh] OR "Ultrasonography"[tiab] OR "intestinal ultrasound"[tiab] OR "bowel ultrasound"[tiab] OR "transabdominal ultrasound"[tiab] OR ultrasound[tiab] OR ultrasonic[tiab] OR sonographic[tiab] OR sonography[tiab] OR SICUS[tiab] OR POCUS[tiab] OR IBUS[tiab] OR IUS[tiab])	**833,866**	**315,429**
**Search 3**	("Prognosis"[Mesh] OR "Prognosis"[tiab] OR "Treatment Outcome"[Mesh] OR "Recurrence"[Mesh] OR "Recurrence"[tiab] OR prognosis[tiab] OR prognostic[tiab] OR predictive[tiab] OR prediction[tiab] OR predict[tiab] OR response[tiab] OR respond[tiab] OR remission[tiab] OR relapse[tiab] OR "loss of response"[tiab] OR discontinuation[tiab] OR persistence[tiab] OR "pre-operative"[tiab] OR "post-operative"[tiab] OR correlation[tiab] OR correlate[tiab])	**8,149,754**	**3,248,987**
**Searches combined**		**1,311**	**301**
**Pubmed and EMBASE combined**		**1612**	

Supplementary Table S3: Intestinal ultrasonography protocols

Study	IUS operator(s) experience	Blinding to ileocolonoscopy findings	Scanning protocol	Interobserver reliability
**DiCandio et al. 1986[1]**	NR	Yes	Preparation: solid and liquid fasting for at least 8 h. Equipment: Toshiba SAL 30A real-time scanner with 2.4–3.5-MHz transducers; suspected areas were reassessed with a short-focus 5-MHz transducer.	NR
**Andreoli et al. 1998[2]**	One sonographer; >2,000 small-bowel sonographic examinations.	Yes	Preparation: overnight fasting; Equipment: Hitachi AU 560 with a 5-MHz convex transducer followed by a 7.5-MHz linear transducer.	NA
**Orlando et al. 2006[3]**	Four operators; approximately 5,000 small-bowel examinations collectively.	NA	Preparation: NR. Equipment: Toshiba Aplio with a 7-MHz probe.	NR
**Rispo et al. 2006[3]**	NR	Yes	Preparation: no special bowel preparation. Equipment: Aloka SSD-1700 with linear and convex 5–7.5-MHz probes.	NR
**Biancone et al. 2007[4]**	One gastroenterologist >2,000 examinations.	Yes	Preparation: 375 mL polyethylene glycol oral contrast. Equipment: Hitachi EUB 6500 with 3.5- and 5-MHz convex and linear-array transducers.	NA
**Castiglione et al. 2008[5]**	NR	Yes	Preparation: no special preparation for IUS; 750 mL polyethylene glycol followed by oral-contrast IUS approximately 30 min later. Equipment: Aloka SSD-1700 with linear and convex 5–7.5-MHz probes.	NR
**Calabrese et al. 2009[6]**	NR	NR	Preparation: 375 mL polyethylene glycol oral contrast (range, 250–500 mL). Equipment: Hitachi EUB 6500 with 3.5- and 5-MHz convex and linear-array transducers.	NR
**Paredes et al. 2010[7]**	NR	Yes	Preparation: fasting. Equipment: Toshiba Aplio 80 with a 3.5-MHz convex or 5–10-MHz linear probe.	NR
**Pallotta et al. 2010[8]**	One sonologist performed SICUS; >9,000 SICUS examinations.	Yes	Preparation: overnight fasting and 375 mL macrogol oral contrast. Equipment: Toshiba Tosbee with 3.5-MHz convex and 5-MHz linear transducers.	NA
**Onali et al. 2010[9]**	One expert gastroenterologist ; >2,000 examinations.	NR	Preparation: 375 mL polyethylene glycol oral contrast; Equipment: Hitachi EUB 6500 with 3.5- and 5-MHz convex and linear-array transducers.	NA

**Paredes et al. 2013[10]**	Two radiologists; ≥15 years' experience in intestinal ultrasonography and ≥5 years' experience in CEUS.	NR	Preparation: NR. Equipment: Toshiba Aplio 80 with 3–6-MHz convex and 6–10-MHz high-frequency probes.	NA
**Onali et al. 2016[11]**	NR	NR	Preparation: fasting; 375 mL polyethylene glycol oral contrast; Equipment: Esaote MyLab Twice with a 1–8-MHz convex transducer and 3–11-MHz high-frequency linear transducer.	NA
**Allocca et al. 2018[12]**	Two independent gastroenterologists; each had ≥6 years' IUS experience.	Yes	Preparation: 6–8 h fasting; Equipment: Aloka Arietta V60 with 1–5-MHz convex and 4–8-MHz microconvex probes.	κ=0.84
**Martínez et al. 2019[13]**	NR	NR	Preparation: 4 h fasting; no oral contrast. Equipment: Toshiba Aplio XG80 with 3.5-MHz convex, 6-MHz curved, and 5–10-MHz linear probes.	NR
**Macedo et al. 2022[14]**	NR	Yes	Preparation: NR. Equipment: HI-VISION Avius with a linear probe using B-mode and Doppler.	NR
**Dragoni et al. 2023[15]**	One sonographer; >6 years' IUS experience.	Yes	Preparation: NR. Equipment: Aloka ProSound α7 with 2–6-MHz convex and 4–13-MHz linear probes.	NR
**Furfaro et al. 2023[16]**	Four bowel-US operators across three centres; each had performed >1,000 examinations over >5 years.	Yes	Preparation: 6–8 h fasting; Equipment: centre-specific Hitachi Arietta 750, Avius Hi Vision, or Noblus systems with 1–6-MHz convex and 4–8-MHz microconvex probes.	NR
**Maconi et al. 2001[17]**	NR	NA	Preparation: NR; Equipment: Ecotron-Aloka SSD-680 real-time scanner.	NR
**Parente et al.2004[18]**	Two operators; >4,000 and >2,000 bowel-US examinations, respectively.	NA	Preparation: overnight fasting; Equipment: Hitachi EUB 525 or Aloka ProSound 5500 SV with a 3.5–6-MHz convex transducer followed by a 7.5–10-MHz linear probe.	NR
**Castiglione et al. 2004[19]**	NR	NA	Preparation: overnight fasting; Equipment: Aloka SSD-1700 with a 3.5–6-MHz convex probe.	NA
**Kunihiro et al. 2007[20]**	NR	NA	Preparation: no special preparation; Equipment: Toshiba SSA-390A with 3–6-MHz curved-array and 6–9-MHz linear-array transducers.	NR
**Rigazio et al. 2009[21]**	Three operators; each performed >200 bowel-US examinations annually	NA	Preparation: 6 h fasting and 500 mL water 30 min before examination. Equipment: Hitachi-Esaote Astro 256 with a 3.5–5-MHz convex probe and 7–10-MHz linear probe.	NR
**Rispo et al. 2018[22]**	NR	Yes	Preparation: overnight fasting; Equipment: LOGIQ S7 with linear and convex 5–9-MHz probes.	NR

**Castiglione et al. 2019[23]**	NR	Yes	Preparation: overnight fasting; Equipment: LOGIQ S7 with linear and convex 5–9-MHz probes.	NR
**Allocca et al. 2022[24]**	Two gastroenterologists; each had ≥7 years' IUS experience.	Yes	Preparation: 6–8 h fasting; no bowel preparation or contrast. Equipment: Aloka Arietta V60 with 1–5-MHz convex and 4–8-MHz microconvex probes.	NR
**Madsen et al. 2025[25]**	Nine IBUS-certified physicians; 0–11 years' experience; 150–700 IUS examinations annually.	NR	Preparation: NR; Equipment: Siemens Sequoia with a 10L4 linear probe at Hvidovre and GE E9/E10 with an L2-9 linear probe at Herlev; an abdominal probe was used when needed.	NR

Supplementary Table S4: Assessment of risk of biases

author et al.	QUADAS-2: Patient selection (Risk of bias)	QUADAS-2: Index test (Risk of bias)	QUADAS-2: Reference standard (Risk of bias)	QUADAS-2: Flow & timing (Risk of bias)	QUADAS-2: Patient selection (Applicability)	QUADAS-2: Index test (Applicability)	QUADAS-2: Reference standard (Applicability)	Overall risk of bias (Low / Some concerns / High) (QUADAS-2)
**Biancone et al. 2007[4]**	Low	Low	Low	Low	Low	Low	Low	Low
**Pallotta et al.2010[8]**	Low	High	Low	Low	Low	Low	Low	Some concerns
**Allocca et al.2018[12]**	Low	Low	Low	Low	Low	Low	Low	Low
**Martínez et al.2019 [13]**	Low	Low	Low	High	Low	Low	Low	Low
**Castiglione et al.2008[5]**	Low	Low	Low	Low	Low	Low	Low	Low
**Andreoli et al. 1998[2]**	unclear	low	low	low	low	low	low	low
**DiCandio et al. 1986[1]**	low	low	unclear	low	low	low	unclear	low
**Rispo et al.2006[26]**	Low	Low	Low	Low	Low	Low	Low	Low
**Paredes et al. 2013[10]**	Low	High	Low	Low	Low	Low	Low	High

**Furfaro et al.2023[16]**	Low	High	Low	High	Low	Low	Low	High
**Dragoni et al.2023[15]**	Low	Low	Low	Low	Low	Low	Low	Low
**Calabrese et al. 2009[6]**	Low	Low	Low	High	Low	Low	Low	Some concerns
**Onali et al. 2010[9]**	Low	Low	Low	High	High	Low	Low	Some concerns
**Paredes et al. 2010[7]**	Low	Low	Low	High	High	Low	Low	High
**Onali et al. 2016[11]**	High	Low	Low	Low	High	Low	Low	Some concerns
**Macedo et al.2022[14]**	Low	Low	Low	Low	Low	Low	Low	Low

Supplementary Table S5: Bias in included studies of prognostic IUS

Prognostic analysis	Participation	Attrition	Prognostic-factor measurement	Outcome measurement	Confounding	Analysis/reporting	overall
**Orlando 2006[3]**	Moderate	Moderate	Low	Low	High	Moderate	High
**Castiglione 2004[19]**	Low	Low	Moderate	Moderate	Moderate	Moderate	Moderate
**Rispo 2018[22]**	Moderate	Low	Low	Moderate	Moderate	Moderate	Moderate
**Madsen 2025[25]**	Low	Low	Low	Moderate	Moderate	High	High
**Rigazio 2009[21]**	High	Low	Low	Low	Moderate	High	High
**Castiglione 2019[23]**	High	Low	Low	Low	Moderate	Moderate	High
**Allocca 2022[24]**	Low	Low	Low	Moderate	Moderate	High	High
**Kunihiro 2007[20]**	Moderate	Low	Moderate	Low	Moderate	Moderate	Moderate
**Maconi 2001[17]**	Moderate	Moderate	Low	Low	Moderate	Moderate	Moderate
**Parente 2004[18]**	Moderate	Low	Low	Moderate	Moderate	Moderate	Moderate

References

1. DiCandio, G.; Mosca, F.; Campatelli, A.; Bianchini, M.; D'Elia, F.; Dellagiovampaola, C.; DiCandio, F.M.G.; Freeman, M.; Vick, C.; Taylor, K.; et al. Sonographic detection of postsurgical recurrence of Crohn disease. *Am. J. Roentgenol.* **1986**, *146*, 523–526. https://doi.org/10.2214/ajr.146.3.523.
2. Andreoli, A.; Cerro, P.; Falasco, G.; Giglio, A.; Prantera, C. Role of Ultrasonography in the Diagnosis of Postsurgical Recurrence of Crohn's Disease. *Am. J. Gastroenterol.* **1998**, *93*, 1117–1121. https://doi.org/10.1111/j.1572-0241.1998.00340.x.
3. Orlando, A.; Modesto, I.; Castiglione, F.; Scala, L.; Scimeca, D.; Rispo, A.; Teresi, S.; Mocciaro, F.; Criscuoli, V.; Marrone, C.; et al. The Role of Calprotectin in Predicting Endoscopic Post-Surgical Recurrence in Asymptomatic Crohn's Disease: A Comparison with Ultrasound. *Eur. Rev. Med. Pharmacol. Sci.* **2006**, *10*, 17–22.
4. Biancone, L.; Calabrese, E.; Petruzziello, C.; Onali, S.; Caruso, A.; Palmieri, G.; Sica, G.S.; Pallone, F. Wireless Capsule Endoscopy and Small Intestine Contrast Ultrasonography in Recurrence of Crohn's Disease. *Inflamm. Bowel Dis.* **2007**, *13*, 1256–1265. https://doi.org/10.1002/ibd.20199.
5. Castiglione, F.; Bucci, L.; Pesce, G.; de Palma, G.D.; Camera, L.; Cipolletta, F.; Testa, A.; Diaferia, M.; Rispo, A. Oral Contrast-Enhanced Sonography for the Diagnosis and Grading of Postsurgical Recurrence of Crohn's Disease. *Inflamm. Bowel Dis.* **2008**, *14*, 1240–1245. https://doi.org/10.1002/ibd.20469.
6. Calabrese, E.; Petruzziello, C.; Onali, S.; Condino, G.; Zorzi, F.; Pallone, F.; Biancone, L. Severity of Postoperative Recurrence in Crohn's Disease: Correlation between Endoscopic and Sonographic Findings. *Inflamm. Bowel Dis.* **2009**, *15*, 1635–1642. https://doi.org/10.1002/ibd.20948.
7. Paredes, J.M.; Ripollés, T.; Cortés, X.; Reyes, M.D.; López, A.; Martínez, M.J.; Moreno-Osset, E. Non-Invasive Diagnosis and Grading of Postsurgical Endoscopic Recurrence in Crohn's Disease. Usefulness of Abdominal Ultrasonography and 99mTc-Hexamethylpropylene Amineoxime-Labelled Leucocyte Scintigraphy. *J. Crohns Colitis* **2010**, *4*, 537–545. https://doi.org/10.1016/j.crohns.2010.03.002.
8. Pallotta, N.; Giovannone, M.; Pezzotti, P.; Gigliozzi, A.; Barberani, F.; Piacentino, D.; Hassan, N.A.; Vincoli, G.; Tosoni, M.; Covotta, A.; et al. Ultrasonographic Detection and Assessment of the Severity of Crohn's Disease Recurrence after Ileal Resection. *BMC Gastroenterol.* **2010**, *10*, 69. https://doi.org/10.1186/1471-230X-10-69.
9. Onali, S.; Calabrese, E.; Petruzziello, C.; Zorzi, F.; Sica, G.S.; Lolli, E.; Ascolani, M.; Condino, G.; Pallone, F.; Biancone, L. Endoscopic vs Ultrasonographic Findings Related to Crohn's Disease Recurrence: A Prospective Longitudinal Study at 3 years. *J. Crohns Colitis* **2010**, *4*, 319–328. https://doi.org/10.1016/j.crohns.2009.12.010.
10. Paredes, J.M.; Ripollés, T.; Cortés, X.; Moreno, N.; Martínez, M.J.; Bustamante-Balén, M.; Delgado, F.; Moreno-Osset, E. Contrast-Enhanced Ultrasonography: Usefulness in the Assessment of Postoperative Recurrence of Crohn's Disease. *J. Crohns Colitis* **2013**, *7*, 192–201. https://doi.org/10.1016/j.crohns.2012.03.017.
11. Onali, S.; Calabrese, E.; Petruzziello, C.; Lolli, E.; Ascolani, M.; Ruffa, A.; Sica, G.; Rossi, A.; Chiaramonte, C.; Pallone, F.; et al. Post-Operative Recurrence of Crohn's Disease: A Prospective Study at 5 Years. *Dig. Liver Dis.* **2016**, *48*, 489–494. https://doi.org/10.1016/j.dld.2016.01.009.
12. Allocca, M.; Fiorino, G.; Bonifacio, C.; Furfaro, F.; Gilardi, D.; Argollo, M.; Peyrin-Biroulet, L.; Danese, S. Comparative Accuracy of Bowel Ultrasound versus Magnetic Resonance Enterography in Combination with Colonoscopy in Assessing Crohn's Disease and Guiding Clinical Decision-Making. *J. Crohns Colitis* **2018**, *12*, 1280–1287. https://doi.org/10.1093/ecco-jcc/jjy093.

13. Martínez, M.J.; Ripollés, T.; Paredes, J.M.; Moreno-Osset, E.; Pazos, J.M.; Blanc, E. Intravenous Contrast-Enhanced Ultrasound for Assessing and Grading Postoperative Recurrence of Crohn's Disease. *Dig. Dis. Sci.* **2019**, *64*, 1640–1650. https://doi.org/10.1007/s10620-018-5432-6.
14. MacEdo, C.P.; Sarmento Costa, M.; Gravito-Soares, E.; Gravito-Soares, M.; Ferreira, A.M.; Portela, F.; Figueiredo, P. Role of Intestinal Ultrasound in the Evaluation of Postsurgical Recurrence in Crohn's Disease: Correlation with Endoscopic Findings. *GE Port. J. Gastroenterol.* **2022**, *29*, 178–186. https://doi.org/10.1159/000517999.
15. Dragoni, G.; Gottin, M.; Innocenti, T.; Lynch, E.N.; Bagnoli, S.; Macrì, G.; Bonanomi, A.G.; Orlandini, B.; Rogai, F.; Milani, S.; et al. Correlation of Ultrasound Scores with Endoscopic Activity in Crohn's Disease: A Prospective Exploratory Study. *J. Crohns Colitis* **2023**, *17*, 1387–1394. https://doi.org/10.1093/ecco-jcc/jjad068.
16. Furfaro, F.; D'Amico, F.; Zilli, A.; Craviotto, V.; Aratari, A.; Bezzio, C.; Spinelli, A.; Gilardi, D.; Radice, S.; Saibeni, S.; et al. Noninvasive Assessment of Postoperative Disease Recurrence in Crohn's Disease: A Multicenter, Prospective Cohort Study on Behalf of the Italian Group for Inflammatory Bowel Disease. *Clin. Gastroenterol. Hepatol.* **2023**, *21*, 3143–3151. https://doi.org/10.1016/j.cgh.2022.11.039.
17. Maconi, G.; Sampietro, G.M.; Cristaldi, M.; Danelli, P.; Russo, A.; Porro, G.B.; Taschieri, A.M. Preoperative Characteristics and Postoperative Behavior of Bowel Wall on Risk of Recurrence After Conservative Surgery in Crohn's Disease A Prospective Study. *Ann. Surg.* **2001**, *233*, 345–352. https://doi.org/10.1097/00000658-200103000-00007.
18. Parente, F.; Sampietro, G.M.; Molteni, M.; Greco, S.; Anderloni, A.; Sposito, C.; Danelli, P.G.; Taschieri, A.M.; Gallus, S.; Bianchi Porro, G. Behaviour of the Bowel Wall during the First Year after Surgery Is a Strong Predictor of Symptomatic Recurrence of Crohn's Disease: A Prospective Study. *Aliment. Pharmacol. Ther.* **2004**, *20*, 959–968. https://doi.org/10.1111/j.1365-2036.2004.02245.x.
19. Castiglione, F.; De Sio, I.; Cozzolino, A.; Rispo, A.; Manguso, F.; Blanco, G.D.V.; Di Girolamo, E.; Castellano, L.; Ciacci, C.; Mazzacca, G. Bowel Wall Thickness at Abdominal Ultrasound and the One-Year-Risk of Surgery in Patients with Crohn's Disease. *Am. J. Gastroenterol.* **2004**, *99*, 1977–1983. https://doi.org/10.1111/j.1572-0241.2004.40267.x.
20. Kunihiro, K.; Hata, J.; Manabe, N.; Mitsuoka, Y.; Tanaka, S.; Haruma, K.; Chayama, K. Predicting the Need for Surgery in Crohn's Disease with Contrast Harmonic Ultrasound. *Scand. J. Gastroenterol.* **2007**, *42*, 577–585. https://doi.org/10.1080/00365520601002716.
21. Rigazio, C.; Ercole, E.; Laudi, C.; Daperno, M.; Lavagna, A.; Crocellà, L.; Bertolino, F.; Viganò, L.; Sostegni, R.; Pera, A.; et al. Abdominal Bowel Ultrasound Can Predict the Risk of Surgery in Crohn's Disease: Proposal of an Ultrasonographic Score. *Scand. J. Gastroenterol.* **2009**, *44*, 585–593. https://doi.org/10.1080/00365520802705992.
22. Rispo, A.; Imperatore, N.; Testa, A.; Bucci, L.; Luglio, G.; De Palma, G.D.; Rea, M.; Nardone, O.M.; Caporaso, N.; Castiglione, F. Combined Endoscopic/Sonographic-Based Risk Matrix Model for Predicting One-Year Risk of Surgery: A Prospective Observational Study of a Tertiary Centre Severe/Refractory Crohn's Disease Cohort. *J. Crohns Colitis* **2018**, *12*, 784–793. https://doi.org/10.1093/ecco-jcc/jjy032.
23. Castiglione, F.; Imperatore, N.; Testa, A.; De Palma, G.D.; Nardone, O.M.; Pellegrini, L.; Caporaso, N.; Rispo, A. One-Year Clinical Outcomes with Biologics in Crohn's Disease: Transmural Healing Compared with Mucosal or No Healing. *Aliment. Pharmacol. Ther.* **2019**, *49*, 1026–1039. https://doi.org/10.1111/apt.15190.
24. Allocca, M.; Craviotto, V.; Bonovas, S.; Furfaro, F.; Zilli, A.; Peyrin-Biroulet, L.; Fiorino, G.; Danese, S. Predictive Value of Bowel Ultrasound in Crohn's Disease: A 12-Month Prospective Study. *Clin. Gastroenterol. Hepatol.* **2022**, *20*, e723–e740. https://doi.org/10.1016/j.cgh.2021.04.029.
25. Madsen, G.R.; Attauabi, M.; Ilvemark, J.F.K.F.; Theede, K.; Bjerrum, J.T.; Bendtsen, F.; Seidelin, J.B.; Wilkens, R.; Boysen, T.; Burisch, J. Intestinal Ultrasound Findings and Their Prognostic Value in Early

Crohn's Disease: A Copenhagen IBD Cohort Study. *Clin. Gastroenterol. Hepatol.* **2025**, *23*, 1398–1407.e6. https://doi.org/10.1016/j.cgh.2024.12.030.

26. Rispo, A.; Bucci, L.; Pesce, G.; Sabbatini, F.; Domenico De Palma, G.; Grassia, R.; Compagna, A.; Testa, A.; Castiglione, F. Bowel Sonography for the Diagnosis and Grading of Postsurgical Recurrence of Crohn_s Disease. *Inflamm. Bowel Dis.* **2006**, *12*, 486–490. https://doi.org/10.1097/00054725-200606000-00007.

Supplementary Figures

Supplementary Figure S1: Flow chart

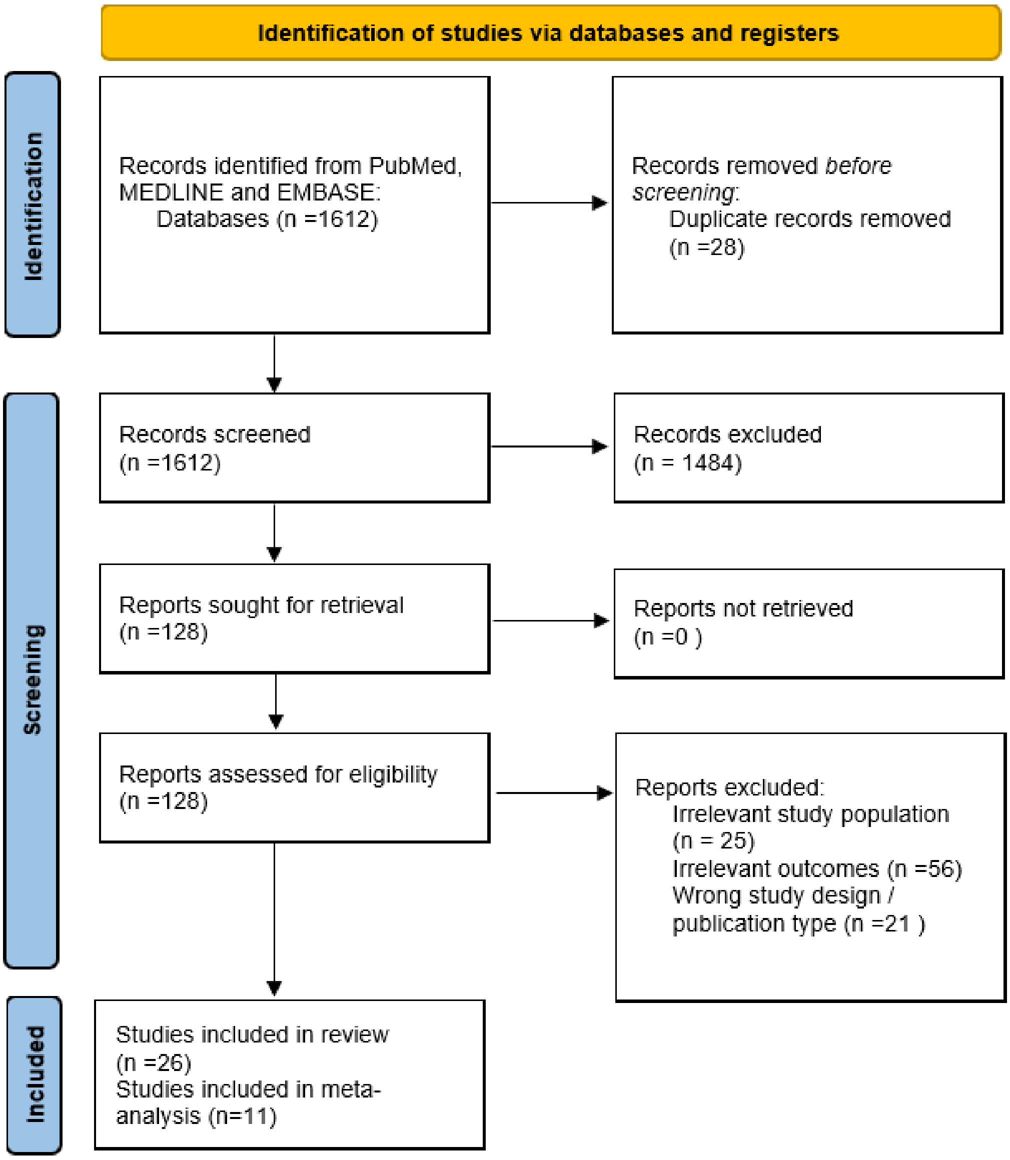

Supplementary Figure S2: Sensitivity analysis with up to 30 days between IUS and endoscopy

Forest plot of log Diagnostic Odds Ratio for post-operative endoscopic recurrence

Study	Index test	TP	FP	FN	TN	Sens	Spec.	Log diagnostic odds ratio
Any postoperative endoscopic recurrence; BWT around 3 mm								
Endoscopic target: Original Rutgeerts >=i1								
Rispo 2006	Conventional B-mode; BWT >3 mm	19	1	5	20	0.79	0.95	4.33 [2.09, 6.57]
Castiglione 2008	Conventional B-mode; maximum BWT >3 mm	17	1	5	17	0.77	0.94	4.06 [1.81, 6.31]
Paredes 2010	BWT >3 mm and/or positive colour Doppler	20	3	6	4	0.77	0.57	1.49 [-0.26, 3.24]
Paredes 2013	BWT >3 mm with positive colour Doppler	44	2	5	9	0.90	0.82	3.68 [1.89, 5.47]
Pooled RE model: Q = 5.46, df = 3, p = .14; I^2 = 45.71%, τ^2 = 0.86								**3.28 [1.93, 4.63]**
Conventional postoperative endoscopic recurrence								
Endoscopic target: Original Rutgeerts >=i2								
Macedo 2022	Conventional B-mode; BWT >=3.9 mm	16	6	2	15	0.89	0.71	3.00 [1.25, 4.74]
**Single study estimate; no pooled heterogeneity statistics**								**3.00 [1.25, 4.74]**
Severe postoperative endoscopic recurrence; BWT around 5 mm								
Endoscopic target: Original Rutgeerts i3/i4								
Rispo 2006	Conventional B-mode; BWT >5 mm	15	0	1	29	0.94	1.00	6.41 [3.15, 9.67]
Paredes 2010	Conventional B-mode; BWT >5 mm	12	4	3	14	0.80	0.78	2.64 [0.96, 4.32]
Paredes 2013	Conventional B-mode; BWT >5 mm	28	6	6	20	0.82	0.77	2.74 [1.48, 4.01]
Pooled RE model: Q = 4.54, df = 2, p = .10; I^2 = 58.70%, τ^2 = 1.27								**3.39 [1.69, 5.08]**
Oral-contrast ultrasound; any endoscopic recurrence								
Endoscopic target: Original Rutgeerts >=i1								
Castiglione 2008	OCBS; maximum BWT >3 mm	18	1	4	17	0.82	0.94	4.34 [2.05, 6.63]
Calabrese 2009	SICUS; BWT >3 mm and/or other findings	62	4	5	1	0.93	0.20	1.13 [-1.24, 3.50]
Biancone 2007	SICUS; BWT >3 mm and/or other findings	21	1	0	0	1.00	0.00	2.66 [-1.59, 6.92]
Onali 2010	SICUS; BWT >3 mm and/or other findings	24	1	0	0	1.00	0.00	2.79 [-1.46, 7.05]
Onali 2016	SICUS; BWT >=3 mm	39	0	0	1	1.00	1.00	5.47 [1.22, 9.71]
Pooled RE model: Q = 3.63, df = 1, p = .06; I^2 = 72.46%, τ^2 = 3.72								**2.75 [-0.39, 5.89]**

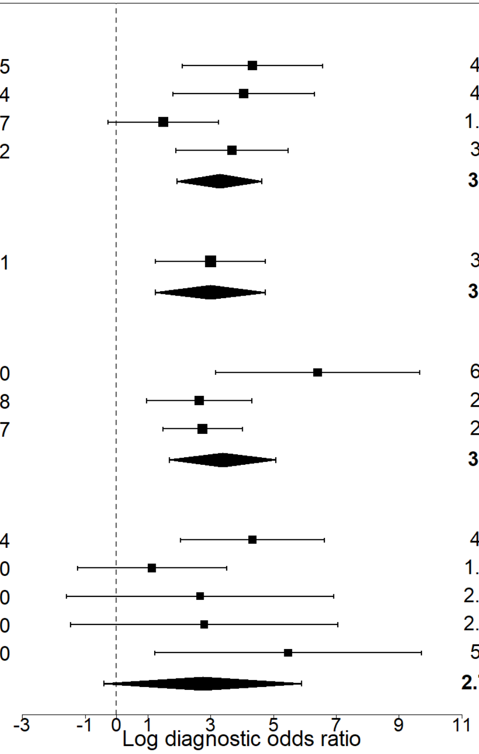

Supplement: Supplementary file 1 [file jcm-15-07024-s001.zip › jcm-4512095-supplementary.pdf]
